# Supplementary material for: Rationale and design of a Scale-Up Project Evaluating Responsiveness to Home Exercise And Lifestyle Tele-Health (SUPER-HEALTH) in people with physical/mobility disabilities: a type 1 hybrid design effectiveness trial
Source: BMJ Open. 2019 Mar 30;9(3):e023538. doi: 10.1136/bmjopen-2018-023538 (PMC6475257; doi:10.1136/bmjopen-2018-023538)
Supplement: Supplementary file 1 [file bmjopen-2018-023538.draft_revisions.pdf]

BMJ Open is committed to open peer review. As part of this commitment we make the peer review history of every article we publish publicly available.

When an article is published we post the peer reviewers' comments and the authors' responses online. We also post the versions of the paper that were used during peer review. These are the versions that the peer review comments apply to.

The versions of the paper that follow are the versions that were submitted during the peer review process. They are not the versions of record or the final published versions. They should not be cited or distributed as the published version of this manuscript.

BMJ Open is an open access journal and the full, final, typeset and author-corrected version of record of the manuscript is available on our site with no access controls, subscription charges or pay-per-view fees (http://bmjopen.bmj.com).

If you have any questions on BMJ Open's open peer review process please email info.bmjopen@bmj.com

BMJ Open

Rationale and Design of a Scale-Up Project Evaluating Responsiveness to Home Exercise and Lifestyle Tele-Health (SUPER-HEALTH) in People with Mobility Disabilities

Journal:	*BMJ Open*
Manuscript ID	bmjopen-2018-023538
Article Type:	Protocol
Date Submitted by the Author:	17-Apr-2018

Complete List of Authors:	Rimmer, James H. ; University of Alabama at Birmingham, UAB/Lakeshore Research Collaborative<br>Mehta, Tapan; University of Alabama at Birmingham, Department of Health Services Administration<br>Wilroy, Jereme; University of Alabama at Birmingham, UAB/Lakeshore Research Collaborative<br>Lai, Byron; University of Alabama at Birmingham, UAB/Lakeshore Research Collaborative<br>Young, Hui-Ju; University of Alabama at Birmingham, UAB/Lakeshore Research Collaborative<br>Kim, Yumi; University of Alabama at Birmingham, UAB/Lakeshore Research Collaborative<br>Pekmezi, D; University of Alabama at Birmingham, Department of Health Behavior<br>Thirumalai, Mohanraj; University of Alabama at Birmingham, Department of Health Services Administration

Keywords:	telerehabilitation, mobility disabilities, telehealth, exercise, disability, mhealth

SCHOLARONE™
Manuscripts

Rationale and design of a Scale-Up Project Evaluating Responsiveness to Home Exercise and Lifestyle Tele-Health (SUPER-HEALTH) in people with mobility disabilities

James Rimmer,[1] PhD, Tapan Mehta,[1] PhD, Jereme Wilroy,[1] PhD, Byron Lai,[1] PhD, Hui-Ju Young,[1] PhD, Yumi Kim,[1] MA, Dorothy Pekmezi,[1] PhD, Mohanraj Thirumalai,[1] PhD

Author affiliations

[1]University of Alabama at Birmingham, Birmingham, USA

Author Contributions

JR provided content for the introduction and discussion. TM provided content for the data analysis and power calculation sections. MT provided all content on app development and social networking section. JW compiled the first full draft of the manuscript, providing content for recruitment section, and made all revisions provided by coauthors. BL provided all content on equipment and technology. HY provided all content for the exercise video section. YK provided content on data management protocol. DP provided content for theoretical framework section. All authors reviewed paper in full and provided revisions on the entire paper.

Keywords: eHealth, Physical Activity, Physically Disabled, mHealth, Telehealth

Word count: 5,384

Corresponding Author:

Mohanraj Thirumalai

1720 2nd Avenue South

Birmingham, AL 35294-0113

mohanraj@uab.edu

205-934-7189

Word Count: 5385

ABSTRACT

Introduction: Rates of physical inactivity among people with physical disabilities are substantially higher than in the general population while access to home-based exercise programs tailored are almost non-existent. Using a theory-driven eHealth platform, an

For peer review only

innovative exercise program referred to as movement-to-music (M2M) will be delivered as a customized, home-based exercise intervention for adults with mobility disabilities.

Methods and analysis: Participants are being recruited for this randomized controlled trial through outpatient clinics at a large rehabilitation center and randomized to one of three groups: a) M2M; b) M2M plus social networking (M2M^{plus}); and c) attention control (AC). The intervention includes a 12-week adoption phase, 12-week transition phase, and 24-week maintenance phase, at which the collection of objective measures on exercise, fitness and self-reported measures on health will be obtained at the start of each phase and at follow-up. The study compares the effectiveness of M2M and M2M^{plus} in increasing physical activity, adherence, fitness, and physical functioning compared to the AC group and examine the mediators and moderators of the treatment effect.

Ethics and dissemination: The Institutional Review Board of The University of Alabama at Birmingham granted full approval: (IRB-160923002). Dissemination of findings will include publication in peer-reviewed journals, presentations at regional, national, and/or international meetings, and the National Center on Health, Physical Activity and Disability (NCHPAD, www.nchpad.org). This scalability study will strengthen our understanding of the potential benefits of eHealth exercise interventions for people with physical disabilities and build upon strategies that aim to recruit larger samples in exercise trials.

Trial registration number: NCT03024320

For peer review only

Strengths and limitations of this study

- The trial is the largest of its kind exploring the use of telehealth technology to improve access to regular and sustainable home exercise among an underserved population of adults with mobility disabilities.

For peer review only - http://bmjopen.bmj.com/site/about/guidelines.xhtml

- The trial addresses a growing need among physicians treating patients with mobility disabilities to have easily accessible e-Health home exercise videos that can be tailored to the functional level of their patients.
- Utilizing constant monitoring technology will allow researchers to know when participants are not engaged in the intervention and prompt coaching calls to reduce dropout.
- Recruitment will occur through a single rehabilitation hospital network and may not be generalizable to other regions across the United States and other countries.

For peer review only

For peer review only - http://bmjopen.bmj.com/site/about/guidelines.xhtml

BACKGROUND

Despite what is known about the positive effects of exercise in improving health and function,[1] people with physical disabilities remain one of the most least active and obese[2-8] populations in society. In the latest data from the Centers for Disease Control and Prevention, nearly one half of all adults with disabilities get no aerobic physical activity.[9] These patterns of low physical activity become more problematic across the lifespan when the effects of the natural aging process are compounded by years of sedentary living, poor nutrition and severe deconditioning.[10-13]

In addition to the physical impairments associated with a disability limiting physical activity, initiating and maintaining a physically active lifestyle is difficult for people with physical disabilities due to a number of barriers faced in the home, built environment (neighborhood, community-based exercise and recreation facilities) and healthcare system.[14] Dozens of papers have reported that the built environment creates substantial limitations in accessing outdoor and indoor physical activity programs and venues.[15-18] Outdoor exercise may be challenging or impossible because many neighborhoods either lack sidewalks or have badly damaged surfaces presenting a high risk of falling among people who have poor balance and/or use wheelchairs.[19] Also, high traffic volume can be intimidating for many people with a mobility disability who have difficulty getting across streets in the time allotted by the traffic light.[20] In terms of getting to an indoor facility such as a fitness center, lack of accessible transportation is often the number one barrier to using these facilities followed by cost of the membership.[20]

Recent data found that people with physical disabilities who were advised by their doctor to exercise were 82 percent more likely to do so.[9] However, the current healthcare system continues to provide little guidance to people with physical disabilities on how to exercise, leaving many, if not most, minimally prepared to manage or improve their health and fitness.[21 22] Therefore, healthcare providers require exercise programs that they can recommend to their

For peer review only - http://bmjopen.bmj.com/site/about/guidelines.xhtml

patients with disabilities that are evidence-based, easily accessible, and tailored to the specific functional and health-related needs of the individual.

In recent years, Internet-based interventions targeting health issues such as nutrition, smoking, physical activity, or multiple health behaviors have become increasingly popular.[23] These interventions have several advantages for people with physical disabilities. In addition to the interactive and convenient nature of Internet-based programs, health professionals can utilize Web-platforms to provide tailored information to individual users and potentially reach large groups of people with physical disabilities at relatively low cost. A notable benefit of eHealth programs for people with physical disabilities includes the ability to participate at home, which is the most convenient place for many people with physical/mobility disabilities to engage in sustained physical activity behavior. If designed properly, home-based exercise allows for prompt feedback, individual tailoring, and continued personalized guidance and social support.[24] Thus, Internet-based exercise programs create an accessible venue for people with physical disabilities to achieve the exercise regimens prescribed by a health professional and can potentially include individuals who do not have access to conventional community-based exercise programs.

To fully address the barriers to accessing exercise, including the need for healthcare providers to have off-the-shelf resources that they can quickly provide to their patients, technology can play a substantial role in the delivery of home-based exercise to people with physical disabilities. Providing exercise opportunities in the comfort of a person's home would increase the opportunity for regular and sustainable exercise that is currently not available in most communities across the U.S. The proposed scale up exercise intervention will enroll a large cohort of adults with mobility disabilities from a university-based outpatient rehabilitation center to determine if an innovative home-based exercise program can achieve improvements in physical activity adherence, health and functional outcomes. The study is referred to as

For peer review only - http://bmjopen.bmj.com/site/about/guidelines.xhtml

SUPER-HEALTH, which stands for *S*cale-*U*p *P*roject *E*valuating *R*esponsiveness to *H*ome *E*xercise *A*nd *L*ifestyle *T*ele-*H*ealth.

This study aims to test the effectiveness of a home-based e-Health exercise program for increasing physical activity among a clinical population of people with mobility disabilities. The intervention, referred to as Movement-2-Music (M2M), is being compared to an enhanced version of M2M that includes social networking (M2M^{plus}). Both of these interventions are compared to an attention control (AC) group. Secondary aims include (1) estimating improvements in health (pain, sleep, quality of life) and physical function (balance, strength, endurance) between M2M and M2M^{plus} groups compared to the AC group; and (2) assessing mediators and moderators of social cognitive theory (self-efficacy, social support, outcome expectancies) and demographic factors (age, race, disability type) of the hypothesized treatment effect to understand for whom and how the intervention is effective.

METHODS

This protocol was approved by the University's Institutional Review Board (IRB-160923002) and is registered with ClinicialTrials.gov (#NCT03024320) as a phase III clinical trial. This paper follows the SPIRIT checklist[25] and conduct and reporting of the trial follows CONSORT guidelines.[26]

Study design

SUPER-HEALTH is a single site, three-arm parallel group randomized controlled trial evaluating the effects of two intervention groups (M2M and M2M^{plus}) compared to AC in people with mobility disabilities, with assessments conducted at four time points: baseline, 12 weeks, 24 weeks, and 48 weeks.

Recruitment

Participants are being recruited from outpatient clinics of a large university-based rehabilitation center through physicians and their staff and by physical mail-outs from January 2018 until the desired sample size is obtained. Supplementary recruitment strategies include

For peer review only - http://bmjopen.bmj.com/site/about/guidelines.xhtml

brochures placed in clinics, society events, newsletters, advertisements, and word of mouth. Additionally, recruitment will include screening more male participants than females.

In order to enhance the likelihood of enrollment through clinician referral,[27] research staff attend periodic meetings with the physicians in the outpatient center. Recruitment from outpatient clinics include: 1) direct referrals from physicians and their staff; 2) indirect referrals through advertisement materials in waiting room; 3) targeted in-person visits by research staff on the day of a patient's clinic appointment; and 4) routine recruitment visits to the waiting room by research staff. Physicians have been instructed to hand out brochures that include the study objectives and contact information. The identification of eligible participants for in-person visits occurs through the review of medical records and clinic appointments using the password-protected hospital database. After potential participants are identified, the recruitment coordinator greets the individuals in the waiting room, provides an overview of the study objectives, and consents individuals interested in participating in the study.

Physical mail-outs are being delivered to potential participants that are identified from the NIH-funded National Center for Biomedical Computing based at Partners HealthCare System: Informatics for Integrating Biology and the Bedside (i2b2) [https://www.i2b2.org/about/index.html]. The i2b2 has been used to identify study cohorts within the outpatient rehabilitation clinics and address research questions by integrating a wide variety of clinical data sources. After an initial search query of participants located within the same state where the study is being conducted, and who attend one of the outpatient clinics, we identified approximately 5,400 people with neuromuscular disorders and musculoskeletal conditions. This list directs the physical mail-outs, which include a flyer that describes the study and a letter co-signed by the chair of department overseeing the clinics and the principal investigator. Mail-outs are being sent in batches of 100 every 2 to 4 weeks and will be increased or decreased depending on the response rate.

For peer review only - http://bmjopen.bmj.com/site/about/guidelines.xhtml

Exclusion criteria

Individuals with a mobility disability are eligible for the study. In order to remain consistent with other studies,[4] this was defined as self-reported difficulty (1) walking (some, much, unable to do) without special equipment use; (2) walking one-quarter of a mile; (3) remaining on feet for more than two hours; (4) taking 10 uninterrupted steps; (5) kneeling, stooping, or crouching; or (5) standing up from an armless straight chair. Individuals who meet the following criteria are not eligible to participate:

- accumulating more than 60 minutes of moderate/vigorous physical activity per week;
- do not report having a diagnosis of a physical/mobility disability;
- not within working age (18 to 64 yrs. of age);
- currently enrolled in a structured exercise program over the past 6 months;
- unable to use upper, lower or both sets of extremities to exercise;
- unable to converse and read English;
- medically unstable to perform home exercise as determined by their physician;
- cognitive impairment that may preclude self-directed daily activities
- no Internet access.

Study Procedures

The study flow diagram includes three phases of the intervention, Adoption (weeks 1 to 12), Transition (weeks 13 to 24), and Maintenance (weeks 25 to 48) (Figure 1). This phased approach allows for gradual adjustment in the dosage of the intervention and the ability to capture changes within and across phases. Details of the interventions offered in each arm are shown in Table 1.

[Insert Figure 1]

[Insert Table 1]

For peer review only - http://bmjopen.bmj.com/site/about/guidelines.xhtml

All data storage is established via the Research Electronic Data Capture (REDCap),[28] an electronic data capture system. When potential participants fill in their information via the study website, it is automatically stored in REDCap. Potential participants' information with other methods of contact (e.g., mail-back, call) is manually entered into REDCap by the recruitment coordinator. The recruitment coordinator reaches out to potential participants within 48-72 hours based on their preferred contact methods (i.e., phone, emails) for more information regarding the study as well as participation eligibility. If eligible, the participant will be consented over the phone and will then receive baseline surveys to be completed online prior to testing visit. Once participants complete the baseline testing they will be randomized.

After the group allocation, participants receive a study designated email address which is uploaded to REDCap. Surveys are automatically sent at the remaining follow-up time points, 12 weeks, 24 weeks, and 48 weeks, to the new email address. There is a 10-day window open before and after the exact date of follow-up data collection based on the initial lab testing date. Participants receive the same email a maximum of five times (1 per day) if they do not complete the survey. When a research staff member notices the missing data after 5 days, the participant is contacted by phone.

Fitness and physical function measures are assessed in a research lab within a state-of-the-art universally designed community health and fitness facility. Transportation is provided to the participants, if needed. After a review of the consent form, the lab staff completes anthropometric measures (height, weight, waist circumference) and vital signs (blood pressure, heart rate). Participant then complete a battery of fitness and physical function measures (see Table 2) while lab staff record results in a data collection packet.

Interventions

After the lab assessments are completed, the technology coordinator provides the participant with the appropriate equipment based on their group assignment and functional level. The equipment includes a tablet (ASUS Zenpad 3s 10), a tablet case (Fintie Folio Stand

For peer review only - http://bmjopen.bmj.com/site/about/guidelines.xhtml

Cover), stylus, Fitbit Charge 2 HR to monitor physical activity, a set of two pound wrist weights (SPRI Thumblock Wrist Weight) and an optional stand (Musician's Gear Folding Music Stand). The technology coordinator then familiarizes participants with the equipment and also guides participants through the tablet app. Equipment instructions cover basic operation (e.g., turning the devices on and off, connecting to Wi-Fi, etc.) and maintenance skills (e.g., cleaning the devices and damage prevention). Regarding app use, participants are carefully guided through the tablet app content and functions. Following these instructions, participants are then asked to independently navigate through the app and view all content.

The tablet app includes the foundational elements required for rapid deployment of the video and textual content, and is password protected to enable detailed usage tracking. The home page of the app provides brief weekly objectives and links to newly received content. Other features of the app include a video and article library, calendar for scheduling, and activity dashboard with Fitbit data. In addition to these features, the M2M^{plus} group's version of the app includes elements that support social interaction. These ancillary features include the ability to private-message other users, leaderboards that display user progress, a user profile, newsfeed, and the ability to comment on and "like" the exercise videos. The app version for the intervention groups (M2M and M2M^{plus}) includes exercise video content, while the AC group will only receive access to the infographics and the Fitbit dashboard.

Educational Resources

Each group receives textual content (i.e., articles, infographics) aimed at improving health through lifestyle changes and tailored to adults with mobility disabilities. This content includes physical activity recommendations, how to maintain activity, developing healthy nutrition habits, ways to reduce stress, and other health-related information. Each group receives this content weekly for 12 weeks (Adoption phase), bi-weekly for the following 12 weeks (Transition phase), and monthly for the last 24 weeks (Maintenance phase).

For peer review only - http://bmjopen.bmj.com/site/about/guidelines.xhtml

Exercise Video Content

Exercise videos include movements that were adapted from an onsite program that has been conducted at a state-of-the-art fitness facility for people with disabilities. The onsite program incorporated an extensive set of movement routines that were choreographed to the functional needs of people with a range of physical disabilities. For the present study, we modified the onsite program into a video-based package that could be performed independently in the home. The goal of the home-based program is to slowly and safely progress participants towards achieving and maintaining 150 minutes of moderate-intensity physical activity per week, which is based on the guidelines provided by the American College of Sports Medicine.[29 30]

The exercise intervention is referred to as Movement-2-Music (M2M). The M2M intervention includes three broad sub-programs or levels that can be arranged by program staff to meet the functional needs of the participant: 1) able to use all four limbs; 2) able to use only the upper limbs (e.g., someone with lower extremity paralysis); and 3) able to use only one side of the body to exercise (e.g., individuals with stroke/hemiparesis). The determination of the level for each participant is based on their baseline physical function assessments. Each M2M session includes four sets of exercise routines: flexibility, muscle strength, cardiorespiratory fitness, and balance. The video ends with a cool down breathing routine. Participants are instructed to complete the assigned M2M exercise videos three times per week. The session duration and exercise intensity is gradually increased throughout the program.

The exercise videos are delivered across the three intervention phases: 1) Adoption, weeks 1-12; 2) Transition, weeks 13-24; and 3) Maintenance, weeks 25-48. During the Adoption phase, one new video is uploaded each week to the eHealth platform. The exercise program begins with a total of 15 minutes of exercise at week 1 and slowly progresses to 90 minutes at week 12. The rationale for starting the program at a low duration is to help participants build confidence and gradually incorporate the exercise program into their daily life. Each week the videos include newly added routines that are guided by an M2M instructor and an actor with a

For peer review only - http://bmjopen.bmj.com/site/about/guidelines.xhtml

disability that matches the functional level of the participants. When a new routine is introduced, the first segment of the video shows the M2M instructor breaking down the routine and explaining each movement pattern (guided exercise portion). In the following week, the guided exercise portion of the same routine is removed from the video so that the participants can perform the routine directly with music. Participants have the ability to view previously completed exercise videos in their video library.

During the Transition and Maintenance phases, one new video is added biweekly (twice a month) and monthly, respectively. The goal of the Transition phase is to progress participants to a goal of 150 minutes of moderate-intensity exercise.[29 30] The Transition phase starts with three 30-minute sessions at week 13 of the intervention (week 1 of this phase) and ends with three 50-minute sessions at week 24 (150 minutes/week). For the Maintenance phase, the session duration remains the same and there are new monthly videos added as an incentive to encourage participants to adhere to the study protocol by using new M2M routines.

Social Networking System

A Social Networking System (SNS) is integrated into the eHealth platform for the M2M^{plus} group. The SNS contains the essential building blocks of social media including (1) identity, (2) sharing, (3) presence, (4) relationships, (5) reputations, (6) groups, and (7) conversations.[31] Additionally, M2M^{plus} participants join a weekly video conference group discussion session led by the project coordinator who has expertise in motivational interviewing strategies. The purposes of these sessions are to stimulate discussion among participants and to encourage them to support each other. Each session incorporates behavioral change strategies that have been modified from previous research with adults with physical disabilities.[32] The sessions focus on topics related to constructs from the Social Cognitive Theory (the theoretical background for this study): social support, overcoming barriers, and preventing relapse.

For peer review only - http://bmjopen.bmj.com/site/about/guidelines.xhtml

Theoretical Framework

The intervention is grounded in the Social Cognitive Theory,[33] which provides a comprehensive, multi-level model of behavioral change emphasizing personal and environmental factors that lead to the adoption and/or maintenance of health promoting behavior.[34] According to the Social Cognitive Theory, health behavior change is based on reciprocal determinism of the interplay of 3 domains: (1) the behavior, which involves performance or mastery by the individual (behavioral capability, self-regulation); (2) personal factors that involve cognitive, affective and biological events (e.g., self-efficacy); and (3) the environmental factors that facilitate or impede change (social and physical environmental factors).[34] Thus, the intervention includes strategies that aim to increase exercise self-efficacy by including adults with mobility disabilities as demonstrators for exercise videos (M2M and M2M^{plus} only); fostering social support through social networking features (e.g., messages, posts, newsfeeds) and group discussion sessions led by research staff (M2M^{plus} only); and teaching participants self-regulation strategies such as goal setting, journaling, and reinforcements using infographics (all groups).

Attention control group

In addition to the Fitbit, the AC group receives a tablet that only includes the educational materials. After the 48-week assessment is completed, participants receive access to the exercise videos.

Randomization

Eligible participants are randomized into one of three arms with 1:1:1 allocation ratio using a permuted block randomization approach where the block size is unknown to the intervention staff. The randomization sequence is generated a priori using a computer-generated random schedule in a permuted block (SAS version 9.4). The randomization schedule is then embedded into a randomization module in REDCap.[28] This system allows

For peer review only - http://bmjopen.bmj.com/site/about/guidelines.xhtml

researchers to manage the information with a higher level of security, remove physical envelop, and set individual level of blinding within the system.

Staff performing recruitment, outcome measurements, and data entry for the primary outcomes are blinded to group allocation. However, due to the nature of the intervention, it is not possible to blind the staff administering interventions or the participants. Participants are instructed not to inform the data collection staff of their intervention status when they return for follow-up measures.

Outcome measures

Lab-based research outcome measures are administered at baseline, 12 weeks, and 48 weeks by independent evaluators blind to treatment assignment. Survey measures are automatically delivered by the REDCap system at baseline, 12 weeks, 24 weeks, and 48 weeks.

The primary outcomes are change in rates of exercise participation and adherence to the study protocol from baseline to transition (24 weeks). There is an additional follow-up at 48 weeks. Exercise data are collected with an activity monitor (Fitbit Charge 2) and stored as total steps per week,[35 36] in addition to a self-report measure, the Godin Leisure-Time Exercise Questionnaire (GLTEQ),[37] at the four time points. Adherence is defined as the percentage the participant meets or exceeds the study protocol's weekly exercise video target minutes, which begins at 15 minutes for the first week and increases approximately 5 minutes each week until reaching 150 minutes of exercise video viewing per week by the end of the transition phase. The 150 minutes per week target will remain throughout the maintenance phase. To improve adherence, the project coordinator monitors weekly data uploaded from the Fitbit and tablet that include the date, total steps per day, and the number of minutes spent viewing videos, respectively. If the Fitbit is inactive for more than three days during the week (7-day period) or if the amount of video viewing does not meet the prescribed dosage for minutes of activity for that week, the participant is contacted by research staff trained in motivational interviewing

For peer review only - http://bmjopen.bmj.com/site/about/guidelines.xhtml

techniques. The purpose of the call is to gather information on non-adherence and aid the participant in creating an action plan for meeting the prescribed activity.

Health and physical function measures include a battery of tests performed at baseline, 12 weeks, and 48 weeks (see Table 2). In addition, anthropometric measures and vital signs are assessed at each testing visit. Short forms developed by the National Institutes of Health (NIH) Patient-Reported Outcome Measurement Information System (PROMIS) are used to measure symptoms and quality of life indicators. The PROMIS short forms used for this study include sleep disturbance, pain, depression, anxiety, physical function, and social interactions. Social cognitive theory measures include surveys and scales on exercise goal-setting, barriers to physical activity, outcome expectations for exercise, social support, and exercise self-efficacy. Other measures include app usability, eHealth literacy assessment, and demographics (i.e., race, sex, age). A brief tabulation of the measures for this study including information on key outcome variables, covariates, and time points is shown in Table 2.

[Insert Table 2]

Power and sample size

A sample size of 648 participants resulted from conducting conservative sample size calculations with minimal assumptions which involved the following assumptions: 80% power, two sided t-test comparing changes in physical activity and adherence, type 1 error rate of 0.025 to account for multiple testing (with two primary outcomes), attrition rate of 0.36, and intent to treat analyses. Each pairwise comparison (M2M versus AC, M2Mplus versus AC and M2M versus M2Mplus) is considered as family of hypothesis. We did not account for multiple comparisons (between different arms) since these are planned a priori. A sample size of 648, which result in 415 participants as completers after accounting for the 36% attrition, provides 80% power to detect an effect size of 0.375 (Cohen's d) between any of the pairwise comparisons aforementioned above.

For peer review only - http://bmjopen.bmj.com/site/about/guidelines.xhtml

Previous effect sizes (ES) estimated for changes in exercise from two web-based interventions reported ES of 0.6129 and 0.8, respectively,[38 39], and ES achieved from two home-based behavioral interventions to increase exercise in wheelchair users above 0.5.[40 41] Our minimum detectable effect size of 0.375 is much smaller than the aforementioned effect sizes to account for the variability in the effect sizes and winner's curse[42] (anticipated scenario given that we aim to recruit a far larger sample size). An assumed attrition rate (AR) of 36% was based on the study by Froehlich-Grobe et al.[41] While our prior studies had an AR as low as 13%, the eHealth literature cautions of higher attrition rates. Achieving lower ARs may possibly relate to the role of health/motivational coaches in the study. Finally, we will be able to bring more efficiency in terms of power and effect size since our primary statistical analysis is based on mixed modeling approach. The mixed modeling approach accounts for correlation between measurements from the same participant that improves that improves power for the same effect size or provides us the ability to detect a lower effect size for the same power and aforementioned assumptions.[42] Mixed modeling technique also utilizes all the data available (that is imputes any missing outcome data). Hence, despite attrition we will have a larger sample size while conducting longitudinal analyses, which would again bring more power/sample efficiency.

Analyses

All statistical analyses are conducted in an intent-to-treat manner, at the individual level. Statistical significance is evaluated at 0.05 levels after adjusting for multiple testing corrections using Bonferroni correction. In general, missingness in outcomes is handled by mixed models for repeated measures data. Quality control (QC) includes descriptive and graphical approaches to summarize baseline characteristics of all key variables.

The primary aim includes two hypotheses stating that the M2M and M2M^{plus} interventions will lead to greater increases in exercise from baseline to the transition phase when compared to the AC group. The outcome variable will be a time-varying measure (two

For peer review only - http://bmjopen.bmj.com/site/about/guidelines.xhtml

time points: baseline and transition phase) of exercise. Using accelerometer (Fitbit) data, total activity of a participant per day will be estimated. The baseline exercise measure will be calculated as the average exercise per day scored across a 7-day period prior to starting the intervention (Adoption phase). Similarly, the average exercise per day for each of the intervention phases will be estimated. The main hypothesis addresses the change in activity between baseline and transition phases across intervention groups. Baseline covariate measures will be included if differences are identified across intervention arms, or if inclusion of these covariates improves the corrected Akaike Information Criterion (AIC).[43 44]

In addition to any baseline covariates, fixed effects will include intervention arms (M2M, M2M^{plus}), AC, Time (baseline, transition) and an Arms X Time interaction. Contrast statements will be used to test the null hypotheses in conjunction with the fitted model coefficients. The estimates of the change in exercise for each arm and their 95% confidence interval will also be reported. Secondary data analyses will include testing for non-linearity in the differences between change scores in exercise across the intervention phases and testing whether there were greater increases in exercise in M2M and/or M2M^{plus} compared to AC from baseline to end of adoption and baseline to transition.

Ethics and dissemination

The IRB will remain informed of any protocol changes or adverse events for the safety of participants. The recruitment and project coordinators will consent each participant to the study. Once the participant signs the informed consent, they receive a unique research ID code and no personally identifiable information will be linked to data collected for this study. Quality assurance procedures are used to minimize missing data, possible errors, and to correct errors before final database lock. After completion of data entry, two additional research staff (project coordinator and data reviewer) audit data periodically and lock the record. When the survey is submitted to REDCap, research staff receive a notification via email and review the data within 48 hours. An independent Data Safety and Monitoring Committee is not necessary due to the

For peer review only - http://bmjopen.bmj.com/site/about/guidelines.xhtml

minimal risk associated with participant outcomes. All data collection will be overseen by the principal investigator and co-investigators, including a biostatistician and physician.

Findings from this study will be shared publicly and disseminated by: (1) publication in peer-reviewed journals; and/or (2) presentations at regional, national, and/or international meetings. Findings will also be disseminated through the National Center on Health, Physical Activity and Disability (NCHPAD, www.nchpad.org), which has over 20,000 email subscribers. If the M2M exercise intervention achieves successful outcomes, we will make the program available to other medical centers through the NCHPAD website.

Data Sharing: We are committed to the sharing of final research data, being mindful that the rights and privacy of our research participants must be protected at all times, that there is the need to protect patentable and other proprietary data (ie, our web-based platform), and that restrictions on data sharing may be imposed by agreements with third parties. Published data with non-identifiers will also be shared with the 2018 Physical Activity Guidelines committee upon request.

Patient and Public Involvement: There was no patient or public involvement in the design of this study.

Competing Interests: No authors have any competing interests.

DISCUSSION

This paper has presented the background and design for a randomized controlled trail investigating the effectiveness of a home-based, eHealth exercise program for adults with mobility disabilities. To our knowledge, this study is the largest exercise trial ever conducted on people with mobility disabilities.

Engaging in the recommended levels of physical activity (150 minutes of moderate intensity physical activity per week) can be extremely challenging for many people with mobility disabilities, who have limited to no access to home exercise equipment, and indoor and outdoor physical activity programs and venues (e.g., outdoor parks, fitness facilities).[15-18 45] In

For peer review only - http://bmjopen.bmj.com/site/about/guidelines.xhtml

particular, outdoor exercise, used by many people who do not have a mobility disability (e.g., walking, jogging, hiking, cycling), may be difficult or impossible to perform by people with mobility disabilities. This is because neighborhoods either lack sidewalks or have surfaces that are badly damaged imposing a risk of falls; high traffic volume makes it cumbersome to get across streets; hilly terrain may be too difficult to traverse; and for people who use wheelchairs, manually pushing a chair for an extended period of time to achieve the recommended levels of exercise may induce or worsen shoulder pain.[46] Indoor exercise at a local fitness center has also been documented as being largely inaccessible to people with mobility disabilities due to lack of accessible exercise equipment and programs and [47 48] transportation. Membership costs often exceed economic resources.[45]

The low level of exercise participation observed in people with mobility disabilities likely have a negative impact on their health. Nearly one-half of people with disabilities are physically inactive, which the U.S. Centers for Disease Control and Prevention defines as not engaging in *any* aerobic physical activity, and this large inactive subgroup is 50 percent more likely to have a chronic disease compared to those who get the recommended amount of aerobic physical activity on a weekly basis.[9] Unfortunately, the current U.S. healthcare system is woefully underprepared to prescribe recommended exercise to people with mobility disabilities, leaving many, if not most, minimally prepared to manage or improve their own health and fitness as they grow older and have more health issues.[21 22 49] Health care providers clearly need more exercise resources that they can recommend to their patients with disabilities that are evidence-based, easily accessible, and tailored to their specific functional level.

The SUPER-HEALTH project addresses this growing epidemic of physical inactivity among people with mobility disabilities by providing simple access to an exercise program tailored to the individuals' functional level and performed in the comfort of their home. Delivery of the exercise program through a tablet app offers opportunities to (a) reach isolated populations who have no other way to obtain regular exercise tailored to their unique needs; (b)

For peer review only - http://bmjopen.bmj.com/site/about/guidelines.xhtml

eliminate transportation difficulties needing to get to a facility-based intervention; and (c) allow the individual to exercise at a time of the day that is convenient for them. Other benefits include their low cost (compared to on-site programs) and the ability for individuals to work at a self-selected pace in a comfortable environment.[24 50]

There are several novel features embedded into SUPER-HEALTH. First, eHealth (telehealth) technology's capacity to provide patient engagement related to health/wellness can create a connected healthcare model that links individuals to trained health/exercise professionals and their healthcare provider offering them greater participation in their own care. Second, with transportation and program costs being two of the most common barriers reported among people with mobility disabilities in terms of obtaining regular exercise,[17 51-54] home-based programs with cloud-based technology and social support hold strong potential for reaching them in their home. Third, the telehealth technology used in this study is complemented by the latest innovation in activity monitoring. The Fitbit is a state-of-the-art accelerometer that provides an objective measure of physical activity during activity monitoring compared to self-report measures. Fourth, the Internet-based home training package (aerobics, strength, flexibility and balance) is provided in the form of music, which offers greater levels of enjoyment and increases its potential for future scalability. The exercise routines can be mixed and matched with new music and movement patterns, which keeps the exercise routines novel. Finally, the use of an eHealth platform has the potential to shift relatively large segments of the population of people with mobility disabilities from inactive to physically active in a sustainable way that extends beyond the intervention timeframe.

CONCLUSION

SUPER-HEALTH is a multi-level scale up exercise eHealth intervention using healthcare providers as the point-of-entry for enrolling patients with mobility disabilities into the study. If SUPER-HEALTH proves efficacious, promotion of health-enhancing exercise for people with mobility disabilities through an eHealth movement-to-music (M2M) intervention (targeted to

For peer review only - http://bmjopen.bmj.com/site/about/guidelines.xhtml

individual functional level) has strong potential to reach large numbers of individuals who are currently not being prescribed a home-based exercise program by their healthcare provider.

Study sponsorship, funding and organization

This work is supported by a grant from the National Institutes of Health, Eunice Kennedy Shriver National Institute of Child Health and Human Development (1R01HD085186). This study is sponsored by the University of Alabama at Birmingham and will be coordinated by the Lakeshore Foundation, Birmingham, Alabama. The study funder will have no role in the conduct or evaluation of the trial, nor the authority over the study design, analysis, interpretation of results, or preparation of manuscripts or abstracts.

For peer review only

For peer review only - http://bmjopen.bmj.com/site/about/guidelines.xhtml

Figure 1. Study Flowchart

For peer review only

For peer review only - http://bmjopen.bmj.com/site/about/guidelines.xhtml

Table 1. SUPER-HEALTH Study Design and Intervention Plan

Intervention Arm	Phase	Health Education Content	M2M Exercise Videos	Social Networking Support
**Attention Control** (Enables estimation of research personnel involvement effect [*within group*])	Adoption	Weekly	None	*Not offered*
	Transition	Biweekly	None	*Not offered*
	Maintenance	Monthly	None	*Not offered*
**M2M** (Enables estimation of home-based M2M [*between group comparison with AC*])	Adoption	Weekly	Weekly new video	*Not offered*
	Transition	Biweekly	Biweekly new video	*Not offered*
	Maintenance	Monthly	Monthly new video	*Not offered*
**M2M^{Plus}** (Enables estimation of social support of M2M^{Plus} [*between group comparison to M2M*])	Adoption	Weekly	Weekly new video	Offered
	Transition	Biweekly	Biweekly new video	Offered
	Maintenance	Monthly	Monthly new video	Offered

M2M = Movement-2-Music; M2M^{Plus} = M2M + Social Networking

For peer review only

Table 2. Measures, Instruments and Time Points

Variables	Instruments	Time Point	Role	Aims
Physical Activity[1]	Fitbit Tracking data	ongoing	Primary Outcome	1,3
	Godin Leisure-Time Exercise Questionnaire[45]	B, A, T, M		
Cardiorespiratory Endurance[1]	Sub-max Arm Ergometer	B, A, M	Secondary Outcome	2
	Spirometry			
Strength[1]	Grip Strength	B, A, M	Secondary Outcome	2
Adherence[1]	Tracking data	ongoing	Primary Outcome	1,3
Secondary health conditions[2]	NIH PROMIS* questionnaires (pain, fatigue, sleep, QoL)	B, A, T, M	Secondary Outcome	2
Social Cognitive Theory Constructs[2]	Exercise Self-Efficacy Scale[53]	B, A, T, M	Mediation	3
	Exercise Goal-setting Scale[54]			
	Multidimensional Outcomes Expectations for Exercise Scale (MOEES)[55]			
	Barriers in Physical Activity Questionnaire – Mobility Impairment[56]			
	Social Provisions Scale[57]			
Demographics[2]	Questionnaire	Baseline	Covariate	1,2,3
Health History[2]	Questionnaire	B, A, M	Covariate	1,2,3
Physical Function[1]	Timed up and go, Repeated chair stands, Timed 25-foot walk	B, A, M	Secondary Outcome	2
Blood Pressure[1]	Blood Pressure Monitor	B, A, M	Covariate	1,2,3
Anthropometrics[1]	Height, Weight, BMI, Waist Circumference	B, A, M	Covariate	1,2,3
App quality & usability	eHealth Literacy Scale (eHEALS)	B, M	Covariate	1,2,3
	Systems Usability Scale (SUS), Mobile Application Rating Scale (MARS)	A		

[1]Measured, [2]Self-Report, B=Baseline, end of A=Adoption, T=Transition, M= Maintenance

For peer review only

For peer review only - http://bmjopen.bmj.com/site/about/guidelines.xhtml

REFERENCES

1. Lai B CK, Vanerbom K, Bickel C Scott, Rimmer JH, Motl RW. Characteristics of adults with neurologic disability recruited for exercise trials: a secondary analysis. *Adapted Physical Activity Quarterly* In Press doi: 10.1123/apaq.2017-0109
2. Chen AY, Kim SE, Houtrow AJ, et al. Prevalence of obesity among children with chronic conditions. *Obesity (Silver Spring, Md)* 2010;18(1):210-3. doi: 10.1038/oby.2009.185
3. Fox MH, Witten MH, Lullo C. Reducing Obesity Among People With Disabilities. *Journal of disability policy studies* 2014;25(3):175-85. doi: 10.1177/1044207313494236
4. Froehlich-Grobe K, Lee J, Washburn RA. Disparities in obesity and related conditions among Americans with disabilities. *American journal of preventive medicine* 2013;45(1):83-90. doi: 10.1016/j.amepre.2013.02.021
5. Hsieh K, Rimmer JH, Heller T. Obesity and associated factors in adults with intellectual disability. *Journal of Intellectual Disability Research* 2014;58(9):851-63. doi: 10.1111/jir.12100
6. Liou TH, Pi-Sunyer FX, Laferrere B. Physical disability and obesity. *Nutrition reviews* 2005;63(10):321-31.
7. Rimmer JH, Wang E. Obesity prevalence among a group of Chicago residents with disabilities. *Arch Phys Med Rehabil* 2005;86(7):1461-4.
8. Rimmer JH, Wang E, Yamaki K, et al. Documenting disparities in obesity and disability. SEDL; Austin, TX:2010. FOCUS Technical Brief No. 24.
9. Carroll DD, Courtney-Long EA, Stevens AC, et al. Vital signs: disability and physical activity--United States, 2009-2012. *MMWR Morb Mortal Wkly Rep* 2014;63(18):407-13.
10. Ravesloot C, Seekins T, Young QR. Health Promotion for People with Chronic Illness and Physical Disabilities: The Connection between Health Psychology and Disability Prevention. *Clinical Pscychology and Psychotherapy.* 1998;5(2):76-85.
11. Rejeski JW, Focht CB. Aging and Physical Disability: On Integrating Group and Individual Counseling with the Promotion of Physical Activity. *Exercise and Sport Sciences Reviews* 2002;30(4):166-70.
12. Rejeski WJ, Brawley LR, Haskell WL. The prevention challenge: an overview of this supplement. *American journal of preventive medicine* 2003;25(3 Suppl 2):107-9.
13. Rimmer JH, Riley B, Creviston T, et al. Exercise training in a predominantly African-American group of stroke survivors. *Medicine and science in sports and exercise* 2000;32(12):1990-6.
14. Martin Ginis KA, Ma JK, Latimer-Cheung AE, et al. A systematic review of review articles addressing factors related to physical activity participation among children and adults with physical disabilities. *Health psychology review* 2016;10(4):478-94. doi: 10.1080/17437199.2016.1198240
15. Becker H, Stuifbergen A. What Makes It So Hard? Barriers to Health Promotion Experienced by People With Multiple Sclerosis and Polio. *Fam Community Health.* 2004;27(1)75-85.
16. Scelza W, Kalpakjian C, Zemper E, et al. Perceived Barriers to Exercise in People with Spinal Cord Injury. *AM J Phys Rehabil.* 2005;84(8):576-83.
17. Phillips M, Flemming N, Tsintzas K. An exploratory study of physical activity and perceived barriers to exercise in ambulant people with neuromuscular disease compared with unaffected controls. *Clinical Rehabilitation* 2009;23(8):746-55. doi: 10.1177/0269215509334838
18. Zalewski K. Exploring Barriers to Remaining Physically Active: A Case Report of a Person with Multiple Sclerosis. *J Neurol Phys Ther.* 2007;31(1):40-5
19. Clarke P, Ailshire JA, Bader M, et al. Mobility Disability and the Urban Built Environment. *American Journal of Epidemiology* 2008;168(5):506-13. doi: 10.1093/aje/kwn185

For peer review only - http://bmjopen.bmj.com/site/about/guidelines.xhtml

20. Vasudevan V, Rimmer JH, Kviz F. Development of the Barriers to Physical Activity Questionnaire for People with Mobility Impairments. *Disability and health journal* 2015;8(4):547-56. doi: 10.1016/j.dhjo.2015.04.007
21. Groah SL, Charlifue S, Tate D, et al. Spinal cord injury and aging: challenges and recommendations for future research. *Am J Phys Med Rehabil* 2012;91(1):80-93. doi: 10.1097/PHM.0b013e31821f70bc
22. Rimmer JH. Exercise and physical activity in persons aging with a physical disability. *Physical medicine and rehabilitation clinics of North America* 2005;16(1):41-56. doi: 10.1016/j.pmr.2004.06.013
23. Webb TL, Joseph J, Yardley L, et al. Using the internet to promote health behavior change: a systematic review and meta-analysis of the impact of theoretical basis, use of behavior change techniques, and mode of delivery on efficacy. *Journal of medical Internet research* 2010;12(1):e4. doi: 10.2196/jmir.1376
24. Cuijpers P, van Straten A, Andersson G. Internet-administered cognitive behavior therapy for health problems: a systematic review. *Journal of Behavioral Medicine* 2008;31(2):169-77. doi: 10.1007/s10865-007-9144-1
25. Chan A, Tetzlaff JM, Altman DG, et al. Spirit 2013 statement: Defining standard protocol items for clinical trials. *Annals of Internal Medicine* 2013;158(3):200-07. doi: 10.7326/0003-4819-158-3-201302050-00583
26. Schulz KF, Altman DG, Moher D. CONSORT 2010 statement: updated guidelines for reporting parallel group randomised trials. *BMJ (Clinical research ed)* 2010;340:c332. doi: 10.1136/bmj.c332
27. Lindblad AS, Zingeser P, Sismanyazici-Navaie N. Incentives and barriers to neurological clinical research participation. *Clinical investigation* 2011;1(12):1663-68. doi: 10.4155/cli.11.153
28. Harris PA, Taylor R, Thielke R, et al. Research Electronic Data Capture (REDCap) - A metadata-driven methodology and workflow process for providing translational research informatics support. *Journal of biomedical informatics* 2009;42(2):377-81. doi: 10.1016/j.jbi.2008.08.010
29. Garber CE, Blissmer B, Deschenes MR, et al. American College of Sports Medicine position stand. Quantity and quality of exercise for developing and maintaining cardiorespiratory, musculoskeletal, and neuromotor fitness in apparently healthy adults: guidance for prescribing exercise. *Medicine and science in sports and exercise* 2011;43(7):1334-59. doi: 10.1249/MSS.0b013e318213fefb
30. Ferguson B. ACSM's Guidelines for Exercise Testing and Prescription 9th Ed. 2014. *The Journal of the Canadian Chiropractic Association* 2014;58(3):328-28.
31. Kietzmann JH, Hermkens K, McCarthy IP, et al. Social media? Get serious! Understanding the functional building blocks of social media. *Business Horizons* 2011;54(3):241-51. doi: https://doi.org/10.1016/j.bushor.2011.01.005
32. Ellis T, Motl RW. Physical activity behavior change in persons with neurologic disorders: overview and examples from Parkinson disease and multiple sclerosis. *Journal of neurologic physical therapy.* 2013;37(2):85-90. doi: 10.1097/NPT.0b013e31829157c0
33. Bandura A. Social foundations of thought and action: a social cognitive theory. Englewood Cliffs, NJ: Prentice Hall 1986.
34. Bandura A. Health promotion by social cognitive means. *Health education & behavior : the official publication of the Society for Public Health Education* 2004;31(2):143-64. doi: 10.1177/1090198104263660
35. Schrader K, Mentis H, Phipps M, et al. What factors predict Fitbit adherence in Stroke and Parkinson disease? (P6.029). *Neurology* 2017;88(16 Supplement)

For peer review only - http://bmjopen.bmj.com/site/about/guidelines.xhtml

36. Gordon R, Bloxham S. Influence of the Fitbit Charge HR on physical activity, aerobic fitness and disability in non-specific back pain participants. *J Sport Med Physical Fitness* 2017;57(12):1669-75. doi: 10.23736/s0022-4707.17.06688-9
37. Godin Leisure-Time Exercise Questionnaire. *Med Sci in Sport Exercise* 1997;29(Supplement):36-38. doi: 10.1097/00005768-199706001-00009
38. Rimmer JH, Rauworth A, Wang E, et al. A randomized controlled trial to increase physical activity and reduce obesity in a predominantly African American group of women with mobility disabilities and severe obesity. *Preventive Medicine* 2009;48(5):473-79. doi: https://doi.org/10.1016/j.ypmed.2009.02.008
39. Rimmer J, Wang E, Pellegrini C, et al. Telehealth Weight Management Intervention for Adults with Physical Disabilities A Randomized Controlled Trial. *Am J Phys Med Rehabil.* 2013;92(12):1084-94.
40. Froehlich-Grobe K, White GW. Promoting physical activity among women with mobility impairments: a randomized controlled trial to assess a home- and community-based intervention. *Arch Phys Med Rehabil.* 2004;85(4):640-48. doi: https://doi.org/10.1016/j.apmr.2003.07.012
41. Froehlich-Grobe K, Lee J, Aaronson L, et al. Exercise for Everyone: A randomized controlled trial of Project Workout On Wheels in promoting exercise among wheelchair users. *Arch Phys Med Rehabil.* 2014;95(1):20-28. doi: 10.1016/j.apmr.2013.07.006
42. Katherine SB, John PAI, Claire M, et al. Power failure: why small sample size undermines the reliability of neuroscience. *Nature Reviews Neuroscience* 2013;14(5):365. doi: 10.1038/nrn3475
43. P Burnham K, R Anderson D. Multimodel Inference: understanding AIC and BIC in Model Selection. *Sociological Methods and Research.* 2004;33(2)261-304.
44. Burnham K AD. Model selection and multimodel inference: A practical information-theoretic approach. 2 ed: Springer-Verlag 2002.
45. Rimmer JH, Riley B, Wang E, et al. Physical activity participation among persons with disabilities: barriers and facilitators. *American journal of preventive medicine* 2004;26(5):419-25. doi: 10.1016/j.amepre.2004.02.002
46. Salisbury SK, Choy NL, Nitz J. Shoulder pain, range of motion, and functional motor skills after acute tetraplegia. *Arch Phys Med Rehabil* 2003;84(10):1480-5.
47. Rimmer JH. The conspicuous absence of people with disabilities in public fitness and recreation facilities: lack of interest or lack of access? *Am J Health Prom.* 2005;19(5):327-9, ii.
48. Rimmer JH, Padalabalanarayanan S, Malone LA, et al. Fitness facilities still lack accessibility for people with disabilities. *Disability and Health Journal* 2017;10(2):214-21. doi: https://doi.org/10.1016/j.dhjo.2016.12.011
49. Cook KF, Molton IR, Jensen MP. Fatigue and aging with a disability. *Arch Phys Med Rehabil* 2011;92(7):1126-33. doi: 10.1016/j.apmr.2011.02.017
50. Miriam W, Martin-Diener E, Bauer G, et al. Comparison of trial participants and open access users of a web-based physical activity intervention regarding adherence, attrition, and repeated participation. *Journal of medical Internet research* 2010;12(1):e3. doi: 10.2196/jmir.1361
51. Jaarsma EA, Dijkstra PU, Geertzen JH, et al. Barriers to and facilitators of sports participation for people with physical disabilities: a systematic review. *Scandinavian journal of medicine & science in sports* 2014;24(6):871-81. doi: 10.1111/sms.12218
52. Rimmer JH, Rubin SS, Braddock D. Barriers to exercise in African American women with physical disabilities. *Arch Phys Med Rehabil* 2000;81(2):182-8.
53. Rimmer JH, Silverman K, Braunschweig C, et al. Feasibility of a health promotion intervention for a group of predominantly African American women with type 2 diabetes. *The Diabetes educator* 2002;28(4):571-80. doi: 10.1177/014572170202800411

For peer review only - http://bmjopen.bmj.com/site/about/guidelines.xhtml

54. Rimmer JH, Wang E, Smith D. Barriers associated with exercise and community access for individuals with stroke. *Journal of rehabilitation research and development* 2008;45(2):315-22.

For peer review only

For peer review only - http://bmjopen.bmj.com/site/about/guidelines.xhtml

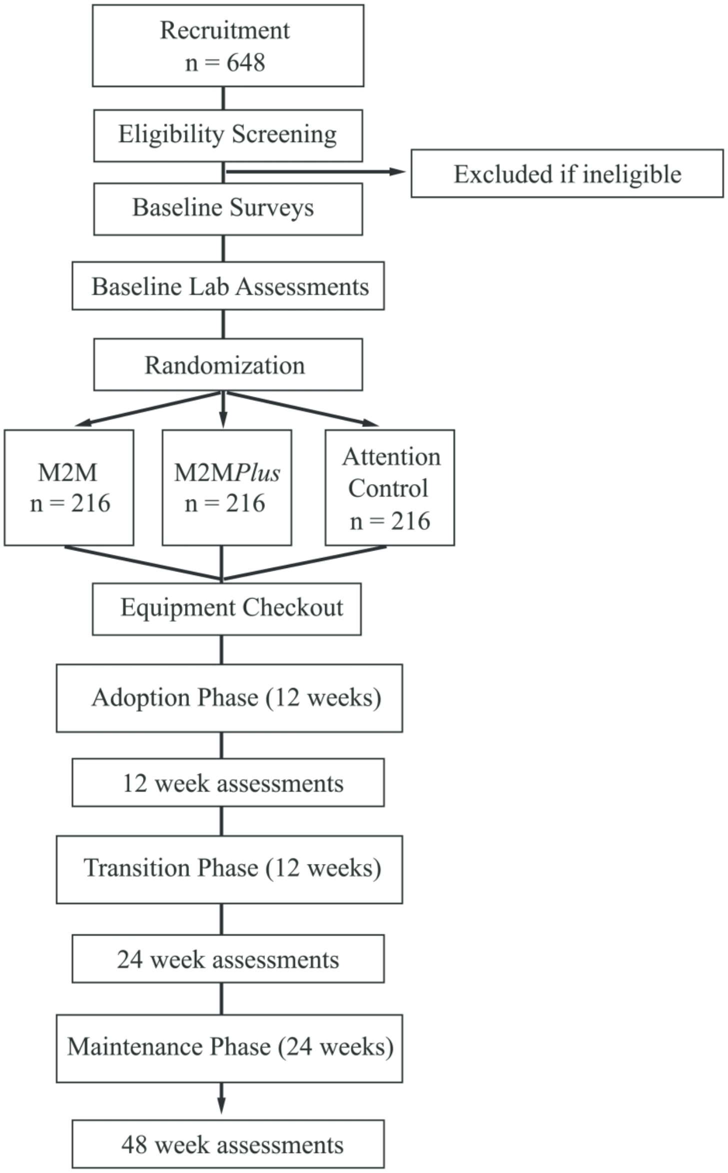

Figure 1. Study Flowchart

224x321mm (300 x 300 DPI)

SPIRIT

STANDARD PROTOCOL ITEMS: RECOMMENDATIONS FOR INTERVENTIONAL TRIALS

SPIRIT 2013 Checklist: Recommended items to address in a clinical trial protocol and related documents*

Section/item	Item No	Description	Addressed on page number
**Administrative information**			
Title	1	Descriptive title identifying the study design, population, interventions, and, if applicable, trial acronym	1
Trial registration	2a	Trial identifier and registry name. If not yet registered, name of intended registry	1
	2b	All items from the World Health Organization Trial Registration Data Set	1
Protocol version	3	Date and version identifier	informed consent
Funding	4	Sources and types of financial, material, and other support	19
Roles and responsibilities	5a	Names, affiliations, and roles of protocol contributors	title page
	5b	Name and contact information for the trial sponsor	19
	5c	Role of study sponsor and funders, if any, in study design; collection, management, analysis, and interpretation of data; writing of the report; and the decision to submit the report for publication, including whether they will have ultimate authority over any of these activities	18
	5d	Composition, roles, and responsibilities of the coordinating centre, steering committee, endpoint adjudication committee, data management team, and other individuals or groups overseeing the trial, if applicable (see Item 21a for data monitoring committee)	n/a

For peer review only

For peer review only - http://bmjopen.bmj.com/site/about/guidelines.xhtml

**Introduction**			
Background and rationale	6a	Description of research question and justification for undertaking the trial, including summary of relevant studies (published and unpublished) examining benefits and harms for each intervention	3-5
	6b	Explanation for choice of comparators	4-5
Objectives	7	Specific objectives or hypotheses	5
Trial design	8	Description of trial design including type of trial (eg, parallel group, crossover, factorial, single group), allocation ratio, and framework (eg, superiority, equivalence, noninferiority, exploratory)	5
**Methods: Participants, interventions, and outcomes**			
Study setting	9	Description of study settings (eg, community clinic, academic hospital) and list of countries where data will be collected. Reference to where list of study sites can be obtained	5-6
Eligibility criteria	10	Inclusion and exclusion criteria for participants. If applicable, eligibility criteria for study centres and individuals who will perform the interventions (eg, surgeons, psychotherapists)	7
Interventions	11a	Interventions for each group with sufficient detail to allow replication, including how and when they will be administered	8-12
	11b	Criteria for discontinuing or modifying allocated interventions for a given trial participant (eg, drug dose change in response to harms, participant request, or improving/worsening disease)	n/a
	11c	Strategies to improve adherence to intervention protocols, and any procedures for monitoring adherence (eg, drug tablet return, laboratory tests)	13
	11d	Relevant concomitant care and interventions that are permitted or prohibited during the trial	7
Outcomes	12	Primary, secondary, and other outcomes, including the specific measurement variable (eg, systolic blood pressure), analysis metric (eg, change from baseline, final value, time to event), method of aggregation (eg, median, proportion), and time point for each outcome. Explanation of the clinical relevance of chosen efficacy and harm outcomes is strongly recommended	13-14; table 2
Participant timeline	13	Time schedule of enrolment, interventions (including any run-ins and washouts), assessments, and visits for participants. A schematic diagram is highly recommended (see Figure)	20

For peer review only - http://bmjopen.bmj.com/site/about/guidelines.xhtml

Sample size	14	Estimated number of participants needed to achieve study objectives and how it was determined, including clinical and statistical assumptions supporting any sample size calculations	14-15
Recruitment	15	Strategies for achieving adequate participant enrolment to reach target sample size	5-6

Methods: Assignment of interventions (for controlled trials)

Allocation:

Sequence generation	16a	Method of generating the allocation sequence (eg, computer-generated random numbers), and list of any factors for stratification. To reduce predictability of a random sequence, details of any planned restriction (eg, blocking) should be provided in a separate document that is unavailable to those who enrol participants or assign interventions	12
Allocation concealment mechanism	16b	Mechanism of implementing the allocation sequence (eg, central telephone; sequentially numbered, opaque, sealed envelopes), describing any steps to conceal the sequence until interventions are assigned	11-12
Implementation	16c	Who will generate the allocation sequence, who will enrol participants, and who will assign participants to interventions	8
Blinding (masking)	17a	Who will be blinded after assignment to interventions (eg, trial participants, care providers, outcome assessors, data analysts), and how	12
	17b	If blinded, circumstances under which unblinding is permissible, and procedure for revealing a participant's allocated intervention during the trial	n/a

Methods: Data collection, management, and analysis

Data collection methods	18a	Plans for assessment and collection of outcome, baseline, and other trial data, including any related processes to promote data quality (eg, duplicate measurements, training of assessors) and a description of study instruments (eg, questionnaires, laboratory tests) along with their reliability and validity, if known. Reference to where data collection forms can be found, if not in the protocol	13-14; table 2
	18b	Plans to promote participant retention and complete follow-up, including list of any outcome data to be collected for participants who discontinue or deviate from intervention protocols	13

For peer review only - http://bmjopen.bmj.com/site/about/guidelines.xhtml

Data management	19	Plans for data entry, coding, security, and storage, including any related processes to promote data quality (eg, double data entry; range checks for data values). Reference to where details of data management procedures can be found, if not in the protocol	8
Statistical methods	20a	Statistical methods for analysing primary and secondary outcomes. Reference to where other details of the statistical analysis plan can be found, if not in the protocol	15-16
	20b	Methods for any additional analyses (eg, subgroup and adjusted analyses)	15-16
	20c	Definition of analysis population relating to protocol non-adherence (eg, as randomised analysis), and any statistical methods to handle missing data (eg, multiple imputation)	15-16
**Methods: Monitoring**			
Data monitoring	21a	Composition of data monitoring committee (DMC); summary of its role and reporting structure; statement of whether it is independent from the sponsor and competing interests; and reference to where further details about its charter can be found, if not in the protocol. Alternatively, an explanation of why a DMC is not needed	16
	21b	Description of any interim analyses and stopping guidelines, including who will have access to these interim results and make the final decision to terminate the trial	n/a
Harms	22	Plans for collecting, assessing, reporting, and managing solicited and spontaneously reported adverse events and other unintended effects of trial interventions or trial conduct	15
Auditing	23	Frequency and procedures for auditing trial conduct, if any, and whether the process will be independent from investigators and the sponsor	17
**Ethics and dissemination**			
Research ethics approval	24	Plans for seeking research ethics committee/institutional review board (REC/IRB) approval	15
Protocol amendments	25	Plans for communicating important protocol modifications (eg, changes to eligibility criteria, outcomes, analyses) to relevant parties (eg, investigators, REC/IRBs, trial participants, trial registries, journals, regulators)	15

For peer review only - http://bmjopen.bmj.com/site/about/guidelines.xhtml

Consent or assent	26a	Who will obtain informed consent or assent from potential trial participants or authorised surrogates, and how (see Item 32)	14
	26b	Additional consent provisions for collection and use of participant data and biological specimens in ancillary studies, if applicable	n/a
Confidentiality	27	How personal information about potential and enrolled participants will be collected, shared, and maintained in order to protect confidentiality before, during, and after the trial	16
Declaration of interests	28	Financial and other competing interests for principal investigators for the overall trial and each study site	19
Access to data	29	Statement of who will have access to the final trial dataset, and disclosure of contractual agreements that limit such access for investigators	16-17
Ancillary and post-trial care	30	Provisions, if any, for ancillary and post-trial care, and for compensation to those who suffer harm from trial participation	informed consent
Dissemination policy	31a	Plans for investigators and sponsor to communicate trial results to participants, healthcare professionals, the public, and other relevant groups (eg, via publication, reporting in results databases, or other data sharing arrangements), including any publication restrictions	16
	31b	Authorship eligibility guidelines and any intended use of professional writers	16
	31c	Plans, if any, for granting public access to the full protocol, participant-level dataset, and statistical code	n/a
**Appendices**			
Informed consent materials	32	Model consent form and other related documentation given to participants and authorised surrogates	appendix
Biological specimens	33	Plans for collection, laboratory evaluation, and storage of biological specimens for genetic or molecular analysis in the current trial and for future use in ancillary studies, if applicable	n/a

*It is strongly recommended that this checklist be read in conjunction with the SPIRIT 2013 Explanation & Elaboration for important clarification on the items. Amendments to the protocol should be tracked and dated. The SPIRIT checklist is copyrighted by the SPIRIT Group under the Creative Commons "Attribution-NonCommercial-NoDerivs 3.0 Unported" license.

For peer review only - http://bmjopen.bmj.com/site/about/guidelines.xhtml

BMJ Open

Rationale and Design of a Scale-Up Project Evaluating Responsiveness to Home Exercise and Lifestyle Tele-Health (SUPER-HEALTH) in People with Mobility Disabilities: A Type 1 Hybrid Design Based Effectiveness Trial

Journal:	*BMJ Open*
Manuscript ID	bmjopen-2018-023538.R1
Article Type:	Protocol
Date Submitted by the Author:	18-Sep-2018
Complete List of Authors:	Rimmer, James H. ; University of Alabama at Birmingham, UAB/Lakeshore Research Collaborative<br>Mehta, Tapan; University of Alabama at Birmingham, Department of Health Services Administration<br>Wilroy, Jereme; University of Alabama at Birmingham, UAB/Lakeshore Research Collaborative<br>Lai, Byron; University of Alabama at Birmingham, UAB/Lakeshore Research Collaborative<br>Young, Hui-Ju; University of Alabama at Birmingham, UAB/Lakeshore Research Collaborative<br>Kim, Yumi; University of Alabama at Birmingham, UAB/Lakeshore Research Collaborative<br>Pekmezi, D; University of Alabama at Birmingham, Department of Health Behavior<br>Thirumalai, Mohanraj; University of Alabama at Birmingham, Department of Health Services Administration
**Primary Subject Heading**:	Rehabilitation medicine
Secondary Subject Heading:	Research methods
Keywords:	telerehabilitation, mobility disabilities, telehealth, exercise, disability, mhealth

SCHOLARONE™
Manuscripts

Rationale and design of a Scale-Up Project Evaluating Responsiveness to Home Exercise and Lifestyle Tele-Health (SUPER-HEALTH) in people with mobility disabilities: A type 1 hybrid design based effectiveness trial

James Rimmer,[1] PhD, Tapan Mehta,[1] PhD, Jereme Wilroy,[1] PhD, Byron Lai,[1] PhD, Hui-Ju Young,[1] PhD, Yumi Kim,[1] MA, Dorothy Pekmezi,[1] PhD, Mohanraj Thirumalai,[1] PhD

Author affiliations

[1]University of Alabama at Birmingham, Birmingham, USA

Corresponding Author:

Mohanraj Thirumalai

1720 2nd Avenue South

Birmingham, AL 35294-0113

mohanraj@uab.edu

205-934-7189

Keywords: eHealth, Physical Activity, Physically Disabled, mHealth, Humans

Word count: 6,643

ABSTRACT

Introduction: Rates of physical inactivity among people with physical disabilities are substantially higher than in the general population while access to home-based exercise programs tailored are almost non-existent. Using a theory-driven eHealth platform, an innovative exercise program referred to as movement-to-music (M2M) will be delivered as a customized, home-based exercise intervention for adults with mobility disabilities.

Methods and analysis: Participants are being recruited for this type 1 hybrid design based effectiveness trial through outpatient clinics at a large rehabilitation center and randomized to one of three groups: a) M2M; b) M2M plus social networking (M2M^{plus}); and c) attention control (AC). The intervention includes a 12-week adoption phase, 12-week transition phase, and 24-week maintenance phase, at which the collection of objective measures on exercise, fitness and

For peer review only

self-reported measures on health will be obtained at the start of each phase and at follow-up. The study compares the effectiveness of M2M and M2M^{plus} in increasing physical activity, adherence, fitness, and physical functioning compared to the AC group and examine the mediators and moderators of the treatment effect.

Ethics and dissemination: The Institutional Review Board of The University of Alabama at Birmingham granted full approval: (IRB-160923002). Dissemination of findings will include publication in peer-reviewed journals, presentations at regional, national, and/or international meetings, and the National Center on Health, Physical Activity and Disability (NCHPAD, www.nchpad.org). This study will strengthen our understanding of the potential benefits of eHealth exercise interventions for people with physical disabilities and build upon strategies that aim to recruit larger samples in exercise trials.

Trial registration number: NCT03024320

Strengths and limitations of this study

- The trial is the largest of its kind exploring the use of telehealth technology to improve access to regular and sustainable home exercise among an underserved population of adults with mobility disabilities.
- The trial addresses a growing need among physicians treating patients with mobility disabilities to have easily accessible e-Health home exercise videos that can be tailored to the functional level of their patients.
- Utilizing constant monitoring technology will allow researchers to know when participants are not engaged in the intervention and prompt coaching calls to reduce dropout.
- Recruitment will occur through a single rehabilitation hospital network and may not be generalizable to other regions across the United States and other countries.

For peer review only

BACKGROUND

Despite what is known about the positive effects of exercise in improving health and function,[1] people with physical disabilities remain one of the least active and obese[2-8] populations in society. In the latest data from the Centers for Disease Control and Prevention, nearly one half of all adults with disabilities get no aerobic physical activity.[9] These patterns of low physical activity become more problematic across the lifespan when the effects of the natural aging process are compounded by years of sedentary living, poor nutrition and severe deconditioning.[10-13]

In addition to the physical impairments associated with a disability limiting physical activity, initiating and maintaining a physically active lifestyle is difficult for people with physical disabilities due to a number of barriers faced in the home, built environment (neighborhood, community-based exercise and recreation facilities) and healthcare system.[14] Dozens of papers have reported that the built environment creates substantial limitations in accessing outdoor and indoor physical activity programs and venues.[15-18] Outdoor exercise may be challenging or impossible because many neighborhoods either lack sidewalks or have badly damaged surfaces presenting a high risk of falling among people who have poor balance and/or use wheelchairs.[19] Also, high traffic volume can be intimidating for many people with a mobility disability who have difficulty getting across streets in the time allotted by the traffic light.[20] In terms of getting to an indoor facility such as a fitness center, lack of accessible transportation is often the number one barrier to using these facilities followed by cost of the membership.[20]

Recent data found that people with physical disabilities who were advised by their doctor to exercise were 82 percent more likely to do so.[9] However, the current healthcare system continues to provide little guidance to people with physical disabilities on how to exercise, leaving many, if not most, minimally prepared to manage or improve their health and fitness.[21 22] Therefore, healthcare providers require exercise programs that they can recommend to their

For peer review only

For peer review only - http://bmjopen.bmj.com/site/about/guidelines.xhtml

patients with disabilities that are evidence-based, easily accessible, and tailored to the specific functional and health-related needs of the individual.

In recent years, Internet-based interventions targeting health issues such as nutrition, smoking, physical activity, or multiple health behaviors have become increasingly popular.[23] These interventions have several advantages for people with physical disabilities. In addition to the interactive and convenient nature of Internet-based programs, health professionals can utilize Web-platforms to provide tailored information to individual users and potentially reach large groups of people with physical disabilities at relatively low cost. A notable benefit of eHealth programs for people with physical disabilities includes the ability to participate at home, which is the most convenient place for many people with physical/mobility disabilities to engage in sustained physical activity behavior. If designed properly, home-based exercise allows for prompt feedback, individual tailoring, and continued personalized guidance and social support.[24] Thus, Internet-based exercise programs create an accessible venue for people with physical disabilities to achieve the exercise regimens prescribed by a health professional and can potentially include individuals who do not have access to conventional community-based exercise programs.

To fully address the barriers to accessing exercise, including the need for healthcare providers to have off-the-shelf resources that they can quickly provide to their patients, technology can play a substantial role in the delivery of home-based exercise to people with physical disabilities. Providing exercise opportunities in the comfort of a person’s home would increase the opportunity for regular and sustainable exercise that is currently not available in most communities across the U.S. The proposed scale up exercise intervention will enroll a large cohort of adults with mobility disabilities from a university-based outpatient rehabilitation center to determine if an innovative home-based exercise program can achieve improvements in physical activity adherence, health and functional outcomes. The study is referred to as

For peer review only - http://bmjopen.bmj.com/site/about/guidelines.xhtml

SUPER-HEALTH, which stands for *S*cale-*U*p *P*roject *E*valuating *R*esponsiveness to *H*ome *E*xercise *A*nd *L*ifestyle *T*ele-*H*ealth.

This study aims to test the effectiveness of a home-based e-Health exercise program for increasing physical activity among a clinical population of people with mobility disabilities. The intervention, referred to as Movement-to-Music (M2M), is being compared to an enhanced version of M2M that includes social networking (M2M^{plus}). Both of these interventions are compared to an attention control (AC) group. Secondary aims include (1) estimating improvements in health (pain, sleep, quality of life) and physical function (balance, strength, endurance) between M2M and M2M^{plus} groups compared to the AC group; and (2) assessing mediators and moderators of social cognitive theory (self-efficacy, self-regulation, social support, outcome expectancies) and demographic factors (age, race, disability type) of the hypothesized treatment effect to understand for whom and how the intervention is effective.

METHODS

This protocol was approved by the University's Institutional Review Board (IRB-160923002) and is registered with ClinicialTrials.gov (#NCT03024320) as a phase III clinical trial. This paper follows the SPIRIT checklist[25], TIDieR checklist[26], and conduct and reporting of the trial follows CONSORT guidelines.[27]

Study design

SUPER-HEALTH is a single site, three-arm parallel group type 1 hybrid design based effectiveness trial[28 29] evaluating the effects of two intervention groups (M2M and M2M^{plus}) compared to AC in people with mobility disabilities, with assessments conducted at four time points: baseline, 12 weeks, 24 weeks, and 48 weeks.

Recruitment

Participants are being recruited from outpatient clinics of a large university-based rehabilitation center through physicians and their staff and by physical mail-outs from January 2018 until the desired sample size is obtained. Supplementary recruitment strategies include

For peer review only - http://bmjopen.bmj.com/site/about/guidelines.xhtml

brochures placed in clinics, society events, newsletters, advertisements, and word of mouth. Additionally, recruitment will include screening more male participants than females.

In order to enhance the likelihood of enrollment through clinician referral,[30] research staff attend periodic meetings with the physicians in the outpatient center. Recruitment from outpatient clinics include: 1) direct referrals from physicians and their staff; 2) indirect referrals through advertisement materials in waiting room; 3) targeted in-person visits by research staff on the day of a patient's clinic appointment; and 4) routine recruitment visits to the waiting room by research staff. Physicians have been instructed to hand out brochures that include the study objectives and contact information. The identification of eligible participants for in-person visits occurs through the review of medical records and clinic appointments using the password-protected hospital database. After potential participants are identified, the recruitment coordinator greets the individuals in the waiting room, provides an overview of the study objectives, and consents individuals interested in participating in the study.

Physical mail-outs are being delivered to potential participants that are identified from the NIH-funded National Center for Biomedical Computing based at Partners HealthCare System: Informatics for Integrating Biology and the Bedside (i2b2) [https://www.i2b2.org/about/index.html]. The i2b2 has been used to identify study cohorts within the outpatient rehabilitation clinics and address research questions by integrating a wide variety of clinical data sources. After an initial search query of participants located within the same state where the study is being conducted, and who attend one of the outpatient clinics, we identified approximately 5,400 people with neuromuscular disorders and musculoskeletal conditions. This list directs the physical mail-outs, which include a flyer that describes the study and a letter co-signed by the chair of department overseeing the clinics and the principal investigator. Mail-outs are being sent in batches of 100 every 2 to 4 weeks and will be increased or decreased depending on the response rate.

For peer review only - http://bmjopen.bmj.com/site/about/guidelines.xhtml

Exclusion criteria

Individuals with a mobility disability are eligible for the study. In order to remain consistent with other studies,[4] this was defined as self-reported difficulty (1) walking (some, much, unable to do) without special equipment use; (2) walking one-quarter of a mile; (3) remaining on feet for more than two hours; (4) taking 10 uninterrupted steps; (5) kneeling, stooping, or crouching; or (5) standing up from an armless straight chair. Individuals who meet the following criteria are not eligible to participate:

- accumulating more than 60 minutes of moderate/vigorous physical activity per week;
- do not report having a diagnosis of a physical/mobility disability;
- not within working age (18 to 64 yrs. of age);
- currently enrolled in a structured exercise program over the past 6 months;
- unable to use upper, lower or both sets of extremities to exercise;
- unable to converse and read English;
- medically unstable to perform home exercise as determined by their physician;
- cognitive impairment that may preclude self-directed daily activities
- no Internet access.

Study Procedures

The study flow diagram includes three phases of the intervention, Adoption (weeks 1 to 12), Transition (weeks 13 to 24), and Maintenance (weeks 25 to 48) (Figure 1). This phased approach allows for gradual adjustment in the dosage of the intervention and the ability to capture changes within and across phases. Details of the interventions offered in each arm are shown in Table 1.

[Insert Figure 1]

[Insert Table 1]

For peer review only - http://bmjopen.bmj.com/site/about/guidelines.xhtml

All data storage is established via the Research Electronic Data Capture (REDCap),[31] an electronic data capture system. When potential participants fill in their information via the study website, it is automatically stored in REDCap. Potential participants' information with other methods of contact (e.g., mail-back, call) is manually entered into REDCap by the recruitment coordinator. The recruitment coordinator reaches out to potential participants within 48-72 hours based on their preferred contact methods (i.e., phone, emails) for more information regarding the study as well as participation eligibility. If eligible, the participant will be consented over the phone and will then receive baseline surveys to be completed online prior to testing visit. Once participants complete the baseline testing they will be randomized.

After the group allocation, participants receive a study designated email address which is uploaded to REDCap. Surveys are automatically sent at the remaining follow-up time points, 12 weeks, 24 weeks, and 48 weeks, to the new email address. There is a 10-day window open before and after the exact date of follow-up data collection based on the initial lab testing date. Participants receive the same email a maximum of five times (1 per day) if they do not complete the survey. When a research staff member notices the missing data after 5 days, the participant is contacted by phone.

Fitness and physical function measures are assessed in a research lab within a state-of-the-art universally designed community health and fitness facility. Transportation is provided to the participants, if needed. After a review of the consent form, the lab staff completes anthropometric measures (height, weight, waist circumference) and vital signs (blood pressure, heart rate). Participant then complete a battery of fitness and physical function measures (see Table 2) while lab staff record results in a data collection packet.

Interventions

After the lab assessments are completed, the technology coordinator provides the participant with the appropriate equipment based on their group assignment and functional level. The equipment includes a tablet (ASUS Zenpad 3s 10), a tablet case (Fintie Folio Stand

For peer review only - http://bmjopen.bmj.com/site/about/guidelines.xhtml

Cover), stylus, Fitbit Charge 2 HR to monitor physical activity, a set of two pound wrist weights (SPRI Thumblock Wrist Weight) and an optional stand (Musician's Gear Folding Music Stand). The technology coordinator then familiarizes participants with the equipment and also guides participants through the tablet app. Equipment instructions cover basic operation (e.g., turning the devices on and off, connecting to Wi-Fi, etc.) and maintenance skills (e.g., cleaning the devices and damage prevention). Regarding app use, participants are carefully guided through the tablet app content and functions. Following these instructions, participants are then asked to independently navigate through the app and view all content.

The tablet app includes the foundational elements required for rapid deployment of the video and textual content, and is password protected to enable detailed usage tracking. The home page of the app provides brief weekly objectives and links to newly received content. Other features of the app include a video and article library, calendar for scheduling, and activity dashboard with Fitbit data. In addition to these features, the M2M^{plus} group's version of the app includes elements that support social interaction. These ancillary features include the ability to private-message other users, leaderboards that display user progress, a user profile, newsfeed, and the ability to comment on and "like" the exercise videos. The app version for the intervention groups (M2M and M2M^{plus}) includes exercise video content, while the AC group will only receive access to the infographics and the Fitbit dashboard.

Educational Resources

Each group receives textual content (i.e., articles, infographics) aimed at improving health through lifestyle changes and tailored to adults with mobility disabilities. This content includes physical activity recommendations, how to maintain activity, developing healthy nutrition habits, ways to reduce stress, and other health-related information. Each group receives this content weekly for 12 weeks (Adoption phase), bi-weekly for the following 12 weeks (Transition phase), and monthly for the last 24 weeks (Maintenance phase).

For peer review only

For peer review only - http://bmjopen.bmj.com/site/about/guidelines.xhtml

Exercise Video Content

Exercise videos include movements that were adapted from an onsite program that has been conducted at a state-of-the-art fitness facility for people with disabilities. The onsite program incorporated an extensive set of movement routines that were choreographed to the functional needs of people with a range of physical disabilities. For the present study, we modified the onsite program into a video-based package that could be performed independently in the home. Program modifications were made using a framework referred to as MAPPETT (*M*odality, *A*daptation, *P*osition, *P*attern, *E*quipment, *T*empo, and *T*ime) to systematically document exercise adaptations. The *M*odality (range of motion/flexibility, muscle strength, cardiorespiratory fitness, and balance), *T*empo (a given range of music beat per minute), and *T*ime (duration of each exercise routine) are intervention elements that are structured and need to be delivered accordingly. The *P*osition (i.e., standing or sitting), *P*attern (i.e., anatomical planes, types of motion, static/dynamic, movement sequence), and *E*quipment (i.e., wrist weight, ankle weight, chair) are elements that can be adapted (*A*daptation) to people with different functional capacities. The goal of the home-based program is to slowly and safely progress participants towards achieving and maintaining 150 minutes of moderate-intensity physical activity per week, which is based on the guidelines provided by the American College of Sports Medicine.[32 33]

The exercise intervention is referred to as M2M. The M2M intervention includes three broad sub-programs or levels that can be arranged by program staff to meet the functional needs of the participant: 1) able to use all four limbs; 2) able to use only the upper limbs (e.g., someone with lower extremity paralysis); and 3) able to use only one side of the body to exercise (e.g., individuals with stroke/hemiparesis). The determination of the level for each participant is based on their baseline physical function assessments. Each M2M session includes four sets of exercise routines: flexibility, muscle strength, cardiorespiratory fitness, and balance. The video ends with a cool down breathing routine. Participants are instructed to

For peer review only - http://bmjopen.bmj.com/site/about/guidelines.xhtml

complete the assigned M2M exercise videos three times per week. The session duration and exercise intensity is gradually increased throughout the program.

The exercise videos are delivered across the three intervention phases: 1) Adoption, weeks 1-12; 2) Transition, weeks 13-24; and 3) Maintenance, weeks 25-48. During the Adoption phase, one new video is uploaded each week to the eHealth platform. The exercise program begins with a total of 15 minutes of exercise at week 1 and slowly progresses to 90 minutes at week 12. The rationale for starting the program at a low duration is to help participants build confidence and gradually incorporate the exercise program into their daily life. Each week the videos include newly added routines that are guided by an M2M instructor and an actor with a disability that matches the functional level of the participants. When a new routine is introduced, the first segment of the video shows the M2M instructor breaking down the routine and explaining each movement pattern (guided exercise portion). In the following week, the guided exercise portion of the same routine is removed from the video so that the participants can perform the routine directly with music. Participants have the ability to view previously completed exercise videos in their video library.

During the Transition and Maintenance phases, one new video is added biweekly (twice a month) and monthly, respectively. The goal of the Transition phase is to progress participants to a goal of 150 minutes of moderate-intensity exercise.[32 33] The Transition phase starts with three 30-minute sessions at week 13 of the intervention (week 1 of this phase) and ends with three 50-minute sessions at week 24 (150 minutes/week). For the Maintenance phase, the session duration remains the same and there are new monthly videos added as an incentive to encourage participants to adhere to the study protocol by using new M2M routines.

Social Networking System

A Social Networking System (SNS) is integrated into the eHealth platform for the M2M^{plus} group. The SNS contains the essential building blocks of social media including (1) identity, (2) sharing, (3) presence, (4) relationships, (5) reputations, (6) groups, and (7) conversations.[34]

For peer review only - http://bmjopen.bmj.com/site/about/guidelines.xhtml

Additionally, M2M^{plus} participants join a weekly video conference group discussion session led by the project coordinator who has expertise in motivational interviewing strategies. The purposes of these sessions are to stimulate discussion among participants and to encourage them to support each other. Each session incorporates behavioral change strategies that have been modified from previous research with adults with physical disabilities.[35] The sessions focus on topics related to constructs from the Social Cognitive Theory (the theoretical background for this study): social support, overcoming barriers, and preventing relapse.

Theoretical Framework

The intervention is grounded in the Social Cognitive Theory,[36] which provides a comprehensive, multi-level model of behavioral change emphasizing personal and environmental factors that lead to the adoption and/or maintenance of health promoting behavior.[37] According to the Social Cognitive Theory, health behavior change is based on reciprocal determinism of the interplay of 3 domains: (1) the behavior, which involves performance or mastery by the individual (behavioral capability, self-regulation); (2) personal factors that involve cognitive, affective and biological events (e.g., self-efficacy); and (3) the environmental factors that facilitate or impede change (social and physical environmental factors).[37] Thus, the intervention targets exercise self-efficacy through self-regulation strategies (incremental goal setting, reinforcements using infographics (all groups) and by including adults with mobility disabilities as demonstrators for exercise videos (M2M and M2M^{plus} only). These approaches focus on mastery experiences, social modeling and verbal persuasion and thus are well aligned with Bandura's research on the key influences on self-efficacy.[38] Social support for physical activity intervention studies among individuals without physical disabilities, such as social networking (e.g., messages, posts, newsfeeds)[39 40] and group discussion sessions[41] led by research staff (M2M^{plus} only).

For peer review only - http://bmjopen.bmj.com/site/about/guidelines.xhtml

Attention control group

In addition to the Fitbit, the AC group receives a tablet that only includes the educational materials. After the 48-week assessment is completed, participants receive access to the exercise videos.

Randomization

Eligible participants are randomized into one of three arms with 1:1:1 allocation ratio using a permuted block randomization approach where the block size is unknown to the intervention staff. The randomization sequence is generated a priori using a computer-generated random schedule in a permuted block (SAS version 9.4). The randomization schedule is then embedded into a randomization module in REDCap.[31] This system allows researchers to manage the information with a higher level of security, remove physical envelop, and set individual level of blinding within the system.

Staff performing recruitment, outcome measurements, and data entry for the primary outcomes are blinded to group allocation. However, due to the nature of the intervention, it is not possible to blind the staff administering interventions or the participants. Participants are instructed not to inform the data collection staff of their intervention status when they return for follow-up measures.

Intervention Fidelity

The intervention uses the latest eHealth technology as a means to deliver and monitor the intervention. Each participant receives the latest in activity monitoring (Fitbit), streaming, content (M2M and M2M^{plus} only), and a social networking platform (M2M^{plus} only), which paired with the use of cloud-based technology allows for the intervention to be administered in the home. Our intervention fidelity plan (Table 2) is based on the *Best Practices and Recommendations from the NIH Behavior Change Consortium (BCC).* The BCC recommendations are intended to link theory and application across five primary study phases,

For peer review only - http://bmjopen.bmj.com/site/about/guidelines.xhtml

including study design, provider training, monitoring and improving the delivery of the intervention, and monitoring and improving the enactment of intervention skills.

[Insert Table 2]

Outcome measures

Lab-based research outcome measures are administered at baseline, 12 weeks, and 48 weeks by independent evaluators blind to treatment assignment. Survey measures are automatically delivered by the REDCap system at baseline, 12 weeks, 24 weeks, and 48 weeks.

The primary outcome is change in rates of exercise participation. Exercise data is collected with a self-report measure, the Godin Leisure-Time Exercise Questionnaire (GLTEQ),[42] at the four time points. A secondary outcome is adherence to the study protocol from baseline to transition (24 weeks), with an additional follow-up at 48 weeks. Adherence is defined as the percentage the participant meets or exceeds the study protocol's weekly exercise session attendance, which is measured by how many times the participant uses the app. The exercise video begins with 5 minutes of exercise, and each participant is asked to complete three times each week equaling a total of 15 minutes for the first week. This increases approximately 5 minutes each week until reaching 150 minutes of exercise video viewing per week (50 minutes of exercise completed three times) by the end of the transition phase. The 150 minutes per week will remain throughout the maintenance phase. To improve adherence and ensure delivery of the intervention, the project coordinator monitors weekly data uploaded from the Fitbit and tablet that include the date, total steps per day, and a log for the exercise videos, which includes a timestamp, title of video, and the number of minutes spent viewing. If there is no Fitbit data for more than seven days or if the amount of logins for video viewing does not meet the prescribed dosage of three times per week, the participant is contacted by research staff trained in motivational interviewing techniques. The purpose of the call is to gather information on non-adherence and aid the participant in creating an action plan for meeting the prescribed activity.

For peer review only - http://bmjopen.bmj.com/site/about/guidelines.xhtml

Other outcomes include health and physical function measures, which involve a battery of tests performed at baseline, 12 weeks, and 48 weeks (see Table 3). In addition, anthropometric measures and vital signs are assessed at each testing visit. Short forms developed by the National Institutes of Health (NIH) Patient-Reported Outcome Measurement Information System (PROMIS) are used to measure symptoms and quality of life indicators. The PROMIS short forms used for this study include sleep disturbance, pain, depression, anxiety, physical function, and social interactions. Social cognitive theory measures include surveys and scales on exercise goal-setting, barriers to physical activity, outcome expectations for exercise, social support, and exercise self-efficacy. Other measures include app usability, eHealth literacy assessment, and demographics (i.e., race, sex, age). A brief tabulation of the measures for this study including information on key outcome variables, covariates, and time points is shown in Table 3.

[Insert Table 3]

Power and sample size

A sample size of 648 participants resulted from conducting conservative sample size calculations with minimal assumptions which involved the following assumptions: 80% power, two sided t-test comparing changes in physical activity, type 1 error rate of 0.025 to account for multiple testing, attrition rate of 0.36, and intent to treat analyses. Each pairwise comparison (M2M versus AC, M2Mplus versus AC and M2M versus M2Mplus) is considered as family of hypothesis. We did not account for multiple comparisons (between different arms) since these are planned a priori. A sample size of 648, which result in 415 participants as completers after accounting for the 36% attrition, provides 80% power to detect an effect size of 0.375 (Cohen's d) between any of the pairwise comparisons aforementioned above.

Previous effect sizes (ES) estimated for changes in exercise from two web-based interventions reported ES of 0.6129 and 0.8, respectively,[43 44], and ES achieved from two home-based behavioral interventions to increase exercise in wheelchair users above 0.5.[45 46] Our

For peer review only - http://bmjopen.bmj.com/site/about/guidelines.xhtml

minimum detectable effect size of 0.375 is much smaller than the aforementioned effect sizes to account for the variability in the effect sizes and winner's curse[47] (anticipated scenario given that we aim to recruit a far larger sample size). An assumed attrition rate (AR) of 36% was based on the study by Froehlich-Grobe et al.[46] While our prior studies had an AR as low as 13%, the eHealth literature cautions of higher attrition rates. Achieving lower ARs may possibly relate to the role of health/motivational coaches in the study. Finally, we will be able to bring more efficiency in terms of power and effect size since our primary statistical analysis is based on mixed modeling approach. The mixed modeling approach accounts for correlation between measurements from the same participant that improves power for the same effect size or provides us the ability to detect a lower effect size for the same power and aforementioned assumptions.[47] Mixed modeling technique also utilizes all the data available (that is imputes any missing outcome data). Hence, despite attrition we will have a larger sample size while conducting longitudinal analyses, which would again bring more power/sample efficiency.

Analyses

All statistical analyses are conducted in an intent-to-treat manner, at the individual level. For the single primary outcome statistical significance will be evaluated at a 2-tailed hypothesis-wise error rate of 0.05. Consistent with published guidelines for statistical reporting, exact p-values (rather than, e.g., "$p < 0.05$" or "NS") will be reported.[48] This allows readers who may have their own opinions about multiple comparison corrections to know the raw uncorrected result and make their own judgments about statistical significance especially given that the significance tests conducted for primary outcome are specified a priori and modest in number. As Saville[49 50] noted, in multiple comparison issues, there is no right answer and each investigator must ultimately "cut the Gordian knot" themselves. For the secondary outcomes statistical significance is evaluated at a family-wise error rate of 0.05 after accounting for multiple outcomes testing. Based on the p-values for all analyses reported, we will also report the false discovery rate and false discovery proportion.

For peer review only - http://bmjopen.bmj.com/site/about/guidelines.xhtml

In general, missingness in outcomes is handled by mixed models for repeated measures data. Quality control (QC) includes descriptive and graphical approaches to summarize baseline characteristics of all key variables.

For the primary outcome of physical activity, we will compare two planned pairwise comparisons. The primary aim includes two hypotheses stating that the M2M and M2M^{plus} interventions will lead to greater increases in exercise from baseline to the transition phase when compared to the AC group. The outcome variable will be a time-varying measure (two time points: baseline and transition phase) of exercise. Using self-report data from the GLTEQ, a Health Contribution Score will be calculated.[42] The baseline exercise measure will be calculated as the average exercise week across a 7-day period prior to starting the intervention (Adoption phase). Similarly, the average exercise for a 7-day period at the end of each intervention phase will be estimated. The main hypothesis addresses the change in activity between baseline and transition phases across intervention groups. Baseline covariate measures will be included if differences are identified across intervention arms, or if inclusion of these covariates improves the corrected Akaike Information Criterion (AIC).[51 52]

In addition to any baseline covariates, fixed effects will include intervention arms (M2M, M2M^{plus}), AC, Time (baseline, transition) and an Arms X Time interaction. Contrast statements will be used to test the null hypotheses in conjunction with the fitted model coefficients. The estimates of the change in exercise for each arm and their 95% confidence interval will also be reported. Secondary data analyses will include testing for non-linearity in the differences between change scores in exercise across the intervention phases and testing whether there were greater increases in exercise in M2M and/or M2M^{plus} compared to AC from baseline to end of adoption and baseline to transition.

Ethics and dissemination

The IRB will remain informed of any protocol changes or adverse events for the safety of participants. The recruitment and project coordinators will consent each participant to the study.

For peer review only - http://bmjopen.bmj.com/site/about/guidelines.xhtml

Once the participant signs the informed consent, they receive a unique research ID code and no personally identifiable information will be linked to data collected for this study. Quality assurance procedures are used to minimize missing data, possible errors, and to correct errors before final database lock. After completion of data entry, two additional research staff (project coordinator and data reviewer) audit data periodically and lock the record. When the survey is submitted to REDCap, research staff receive a notification via email and review the data within 48 hours. An independent Data Safety and Monitoring Committee is not necessary due to the minimal risk associated with participant outcomes. All data collection will be overseen by the principal investigator and co-investigators, including a biostatistician and physician.

Findings from this study will be shared publicly and disseminated by: (1) publication in peer-reviewed journals; and/or (2) presentations at regional, national, and/or international meetings. Findings will also be disseminated through the National Center on Health, Physical Activity and Disability (NCHPAD, www.nchpad.org), which has over 20,000 email subscribers. If the M2M exercise intervention achieves successful outcomes, we will make the program available to other medical centers through the NCHPAD website. We are committed to the sharing of final research data, being mindful that the rights and privacy of our research participants must be protected at all times, that there is the need to protect patentable and other proprietary data (ie, our web-based platform), and that restrictions on data sharing may be imposed by agreements with third parties. Published data with non-identifiers will also be shared with the 2018 Physical Activity Guidelines committee upon request.

DISCUSSION

This paper has presented the background and design for a randomized controlled trail investigating the effectiveness of a home-based, eHealth exercise program for adults with mobility disabilities. To our knowledge, this study is the largest exercise trial ever conducted on people with mobility disabilities.

For peer review only - http://bmjopen.bmj.com/site/about/guidelines.xhtml

Engaging in the recommended levels of physical activity (150 minutes of moderate intensity physical activity per week) can be extremely challenging for many people with mobility disabilities, who have limited to no access to home exercise equipment, and indoor and outdoor physical activity programs and venues (e.g., outdoor parks, fitness facilities).[15-18 53] In particular, outdoor exercise, used by many people who do not have a mobility disability (e.g., walking, jogging, hiking, cycling), may be difficult or impossible to perform by people with mobility disabilities. This is because neighborhoods either lack sidewalks or have surfaces that are badly damaged imposing a risk of falls; high traffic volume makes it cumbersome to get across streets; hilly terrain may be too difficult to traverse; and for people who use wheelchairs, manually pushing a chair for an extended period of time to achieve the recommended levels of exercise may induce or worsen shoulder pain.[54] Indoor exercise at a local fitness center has also been documented as being largely inaccessible to people with mobility disabilities due to lack of accessible exercise equipment and programs and [55 56] transportation. Membership costs often exceed economic resources.[53]

The low level of exercise participation observed in people with mobility disabilities likely have a negative impact on their health. Nearly one-half of people with disabilities are physically inactive, which the U.S. Centers for Disease Control and Prevention defines as not engaging in *any* aerobic physical activity, and this large inactive subgroup is 50 percent more likely to have a chronic disease compared to those who get the recommended amount of aerobic physical activity on a weekly basis.[9] Unfortunately, the current U.S. healthcare system is woefully underprepared to prescribe recommended exercise to people with mobility disabilities, leaving many, if not most, minimally prepared to manage or improve their own health and fitness as they grow older and have more health issues.[21 22 57] Health care providers clearly need more exercise resources that they can recommend to their patients with disabilities that are evidence-based, easily accessible, and tailored to their specific functional level.

For peer review only - http://bmjopen.bmj.com/site/about/guidelines.xhtml

The SUPER-HEALTH project addresses this growing epidemic of physical inactivity among people with mobility disabilities by providing simple access to an exercise program tailored to the individuals' functional level and performed in the comfort of their home. Delivery of the exercise program through a tablet app offers opportunities to (a) reach isolated populations who have no other way to obtain regular exercise tailored to their unique needs; (b) eliminate transportation difficulties needing to get to a facility-based intervention; and (c) allow the individual to exercise at a time of the day that is convenient for them. Other benefits include their low cost (compared to on-site programs) and the ability for individuals to work at a self-selected pace in a comfortable environment.[24 58]

There are several novel features embedded into SUPER-HEALTH. First, eHealth (telehealth) technology's capacity to provide patient engagement related to health/wellness can create a connected healthcare model that links individuals to trained health/exercise professionals and their healthcare provider offering them greater participation in their own care. Second, with transportation and program costs being two of the most common barriers reported among people with mobility disabilities in terms of obtaining regular exercise,[17 59-62] home-based programs with cloud-based technology and social support hold strong potential for reaching them in their home. Third, the telehealth technology used in this study is complemented by the latest innovation in activity monitoring. The Fitbit is a state-of-the-art accelerometer that provides an objective measure of physical activity allowing the participant to receive activity monitoring throughout the program . Fourth, the Internet-based home training package (aerobics, strength, flexibility and balance) is provided in the form of music, which offers greater levels of enjoyment and increases its potential for future scalability. The exercise routines can be mixed and matched with new music and movement patterns, which keeps the exercise routines novel. Finally, the use of an eHealth platform has the potential to shift relatively large segments of the population of people with mobility disabilities from inactive to physically active in a sustainable way that extends beyond the intervention timeframe.

For peer review only - http://bmjopen.bmj.com/site/about/guidelines.xhtml

CONCLUSION

SUPER-HEALTH is a multi-level scale up exercise eHealth intervention using healthcare providers as the point-of-entry for enrolling patients with mobility disabilities into the study. If SUPER-HEALTH proves efficacious, promotion of health-enhancing exercise for people with mobility disabilities through an eHealth movement-to-music (M2M) intervention (targeted to individual functional level) has strong potential to reach large numbers of individuals who are currently not being prescribed a home-based exercise program by their healthcare provider.

For peer review only

For peer review only - http://bmjopen.bmj.com/site/about/guidelines.xhtml

Figure 1. Study Flowchart

For peer review only

For peer review only - http://bmjopen.bmj.com/site/about/guidelines.xhtml

Table 1. SUPER-HEALTH Study Design and Intervention Plan

Intervention Arm	Phase	Health Education Content	M2M Exercise Videos	Social Networking Support
**Attention Control** (Enables estimation of research personnel involvement effect [*within group*])	Adoption	Weekly	None	*Not offered*
	Transition	Biweekly	None	*Not offered*
	Maintenance	Monthly	None	*Not offered*
**M2M** (Enables estimation of home-based M2M [*between group comparison with AC*])	Adoption	Weekly	Weekly new video	*Not offered*
	Transition	Biweekly	Biweekly new video	*Not offered*
	Maintenance	Monthly	Monthly new video	*Not offered*
**$M2M^{Plus}$** (Enables estimation of social support of $M2M^{Plus}$ [*between group comparison to M2M*])	Adoption	Weekly	Weekly new video	Offered
	Transition	Biweekly	Biweekly new video	Offered
	Maintenance	Monthly	Monthly new video	Offered

M2M = Movement-2-Music; $M2M^{Plus}$ = M2M + Social Networking

For peer review only

For peer review only - http://bmjopen.bmj.com/site/about/guidelines.xhtml

Table 2. Intervention Fidelity Plan

Treatment Fidelity Element	Strategies
Study Design	Ensure intervention consistency with SCT.
	Protocol for eHealth platform maintenance and resolution of technical problems during study.
	Weekly delivery and notification of M2M videos to motivate participants to use.
	Phone coaching would assess the use of the eHealth platform and motivate intervention participants to complete their sessions.
	Password protection to ensure that participants access only material belonging to their study arm.
	Wearing of Fitbit will be monitored and if no data showing for 7 days, the participant will be contacted by phone and/or email.
	Intervention delivered via eHealth platform, not via clinicians.
Provider Training	Training of all individuals who will be conducting the screening, phone coaching, and interviews. Manual of Operating procedure to contain standardized scripts for all interactions. Booster training sessions conducted every six months.
	Intervention will be delivered through eHealth platform in exactly the same way to all participants of the two intervention arms.
Delivery of Intervention	Prescribed behaviors (aerobic/strength moves), as defined by the M2M protocol, performed by the interventionist in M2M videos will be qualitatively and quantitatively assessed.
	Technology acceptance will be measured through perceived usefulness and ease of use scale that will provide information about the usability and the extent to which the participant has positive perceptions and intentions to use the platform.
	The research coordinator will regularly monitor the eHealth platform throughout the study to ensure videos and other materials were working correctly.
	Technical difficulties with the delivery of the intervention will be resolved in timely manner.
	The number of participants logins, clicks to video links and minutes watched will be monitored by tools on the intervention eHealth platform server.
Receipt of Intervention	Every time a participant views a M2M video, a timestamp and minutes viewed are recorded.
	Each time the participant reads an articles or opens a video, they have a check in button they press.

For peer review only - http://bmjopen.bmj.com/site/about/guidelines.xhtml

Table 3. Measures, Instruments and Time Points

Variables	Instruments	Time Point	Role
Physical Activity[1]	Godin Leisure-Time Exercise Questionnaire[41]	B, A, T, M	Primary Outcome
Cardiorespiratory Endurance[1]	Sub-max Arm Ergometer<br>Spirometry	B, A, M	Secondary Outcome
Strength[1]	Grip Strength	B, A, M	Secondary Outcome
Adherence[1]	Tracking data	ongoing	Secondary Outcome
Secondary health conditions[2]	NIH PROMIS* questionnaires (pain, fatigue, sleep, QoL)	B, A, T, M	Secondary Outcome
Social Cognitive Theory Constructs[2]	Exercise Self-Efficacy Scale[53]<br>Exercise Goal-setting Scale[54]<br>Multidimensional Outcomes Expectations for Exercise Scale (MOEES)[55]<br>Barriers in Physical Activity Questionnaire – Mobility Impairment[56]<br>Social Provisions Scale[57]	B, A, T, M	Mediation
Demographics[2]	Questionnaire	Baseline	Covariate
Health History[2]	Questionnaire	B, A, M	Covariate
Physical Function[1]	Timed up and go, Repeated chair stands, Timed 25-foot walk	B, A, M	Secondary Outcome
Blood Pressure[1]	Blood Pressure Monitor	B, A, M	Covariate
Anthropometrics[1]	Height, Weight, BMI, Waist Circumference	B, A, M	Covariate
App quality & usability	eHealth Literacy Scale (eHEALS)<br>Systems Usability Scale (SUS), Mobile Application Rating Scale (MARS)	B, M<br>A	Covariate

For peer review only

Author Contributions

JR provided content for the introduction and discussion. TM provided content for the data analysis and power calculation sections. MT provided all content on app development and social networking section. JW compiled the first full draft of the manuscript, providing content for recruitment section, and made all revisions provided by coauthors. BL provided all content on equipment and technology. HY provided all content for the exercise video section. YK provided content on data management protocol. DP provided content for theoretical framework section. All authors reviewed paper in full and provided revisions on the entire paper.

Study sponsorship, funding and organization

This work is supported by a grant from the National Institutes of Health, Eunice Kennedy Shriver National Institute of Child Health and Human Development (1R01HD085186). This study is sponsored by the University of Alabama at Birmingham and will be coordinated by the Lakeshore Foundation, Birmingham, Alabama. The study funder will have no role in the conduct or evaluation of the trial, nor the authority over the study design, analysis, interpretation of results, or preparation of manuscripts or abstracts.

Data Sharing, Patient and Public Involvement, and Competing Interests

We are committed to the sharing of final research data, being mindful that the rights and privacy of our research participants must be protected at all times, that there is the need to protect patentable and other proprietary data (ie, our web-based platform), and that restrictions on data sharing may be imposed by agreements with third parties. Published data with non-identifiers will also be shared with the 2018 Physical Activity Guidelines committee upon request. There was no patient or public involvement in the design of this study. No authors have any competing interests.

For peer review only - http://bmjopen.bmj.com/site/about/guidelines.xhtml

REFERENCES

1. Lai B CK, Vanerbom K, Bickel C Scott, Rimmer JH, Motl RW. Characteristics of adults with neurologic disability recruited for exercise trials: a secondary analysis. *Adapted Physical Activity Quarterly* In Press doi: 10.1123/apaq.2017-0109
2. Chen AY, Kim SE, Houtrow AJ, et al. Prevalence of obesity among children with chronic conditions. *Obesity (Silver Spring, Md)* 2010;18(1):210-3. doi: 10.1038/oby.2009.185 [published Online First: 2009/06/13]
3. Fox MH, Witten MH, Lullo C. Reducing Obesity Among People With Disabilities. *Journal of disability policy studies* 2014;25(3):175-85. doi: 10.1177/1044207313494236
4. Froehlich-Grobe K, Lee J, Washburn RA. Disparities in obesity and related conditions among Americans with disabilities. *Am J Prev Med* 2013;45(1):83-90. doi: 10.1016/j.amepre.2013.02.021 [published Online First: 2013/06/26]
5. Hsieh K, Rimmer JH, Heller T. Obesity and associated factors in adults with intellectual disability. *Journal of Intellectual Disability Research* 2014;58(9):851-63. doi: 10.1111/jir.12100
6. Liou TH, Pi-Sunyer FX, Laferrere B. Physical disability and obesity. *Nutrition reviews* 2005;63(10):321-31. [published Online First: 2005/11/22]
7. Rimmer JH, Wang E. Obesity prevalence among a group of Chicago residents with disabilities. *Arch Phys Med Rehabil* 2005;86(7):1461-4. [published Online First: 2005/07/09]
8. Rimmer JH, Wang E, Yamaki K, et al. Documenting disparities in obesity and disability2010.
9. Carroll DD, Courtney-Long EA, Stevens AC, et al. Vital signs: disability and physical activity--United States, 2009-2012. *MMWR Morb Mortal Wkly Rep* 2014;63(18):407-13. [published Online First: 2014/05/09]
10. Ravesloot C, Seekins T, Young QR. Health Promotion for People with Chronic Illness and Physical Disabilities: The Connection between Health Psychology and Disability Prevention1998.
11. Rejeski JW, Focht CB. Aging and Physical Disability: On Integrating Group and Individual Counseling with the Promotion of Physical Activity. *Exercise and Sport Sciences Reviews* 2002;30(4):166-70.
12. Rejeski WJ, Brawley LR, Haskell WL. The prevention challenge: an overview of this supplement. *Am J Prev Med* 2003;25(3 Suppl 2):107-9. [published Online First: 2003/10/14]
13. Rimmer JH, Riley B, Creviston T, et al. Exercise training in a predominantly African-American group of stroke survivors. *Medicine and science in sports and exercise* 2000;32(12):1990-6. [published Online First: 2000/12/29]
14. Martin Ginis KA, Ma JK, Latimer-Cheung AE, et al. A systematic review of review articles addressing factors related to physical activity participation among children and adults with physical disabilities. *Health psychology review* 2016;10(4):478-94. doi: 10.1080/17437199.2016.1198240 [published Online First: 2016/10/30]
15. Becker H, Stuifbergen A. What Makes It So Hard? Barriers to Health Promotion Experienced by People With Multiple Sclerosis and Polio2004.
16. Scelza W, Kalpakjian C, Zemper E, et al. Perceived Barriers to Exercise in People with Spinal Cord Injury2005.
17. Phillips M, Flemming N, Tsintzas K. An exploratory study of physical activity and perceived barriers to exercise in ambulant people with neuromuscular disease compared with unaffected controls. *Clinical Rehabilitation* 2009;23(8):746-55. doi: 10.1177/0269215509334838
18. Zalewski K. Exploring Barriers to Remaining Physically Active: A Case Report of a Person with Multiple Sclerosis2007.
19. Clarke P, Ailshire JA, Bader M, et al. Mobility Disability and the Urban Built Environment. *American Journal of Epidemiology* 2008;168(5):506-13. doi: 10.1093/aje/kwn185

For peer review only - http://bmjopen.bmj.com/site/about/guidelines.xhtml

20. Vasudevan V, Rimmer JH, Kviz F. Development of the Barriers to Physical Activity Questionnaire for People with Mobility Impairments. *Disability and health journal* 2015;8(4):547-56. doi: 10.1016/j.dhjo.2015.04.007
21. Groah SL, Charlifue S, Tate D, et al. Spinal cord injury and aging: challenges and recommendations for future research. *Am J Phys Med Rehabil* 2012;91(1):80-93. doi: 10.1097/PHM.0b013e31821f70bc [published Online First: 2011/06/18]
22. Rimmer JH. Exercise and physical activity in persons aging with a physical disability. *Physical medicine and rehabilitation clinics of North America* 2005;16(1):41-56. doi: 10.1016/j.pmr.2004.06.013 [published Online First: 2004/11/25]
23. Webb TL, Joseph J, Yardley L, et al. Using the internet to promote health behavior change: a systematic review and meta-analysis of the impact of theoretical basis, use of behavior change techniques, and mode of delivery on efficacy. *Journal of medical Internet research* 2010;12(1):e4. doi: 10.2196/jmir.1376 [published Online First: 2010/02/19]
24. Cuijpers P, van Straten A, Andersson G. Internet-administered cognitive behavior therapy for health problems: a systematic review. *Journal of Behavioral Medicine* 2008;31(2):169-77. doi: 10.1007/s10865-007-9144-1
25. Chan A, Tetzlaff JM, Altman DG, et al. Spirit 2013 statement: Defining standard protocol items for clinical trials. *Annals of Internal Medicine* 2013;158(3):200-07. doi: 10.7326/0003-4819-158-3-201302050-00583
26. Hoffmann TC, Glasziou PP, Boutron I, et al. Better reporting of interventions: template for intervention description and replication (TIDieR) checklist and guide. *BMJ (Clinical research ed)* 2014;348:g1687. doi: 10.1136/bmj.g1687 [published Online First: 2014/03/13]
27. Schulz KF, Altman DG, Moher D. CONSORT 2010 statement: updated guidelines for reporting parallel group randomised trials. *BMJ (Clinical research ed)* 2010;340:c332. doi: 10.1136/bmj.c332 [published Online First: 2010/03/25]
28. Bernet AC, Willens DE, Bauer MS. Effectiveness-implementation hybrid designs: implications for quality improvement science. *Implementation Science : IS* 2013;8(Suppl 1):S2-S2. doi: 10.1186/1748-5908-8-S1-S2
29. Curran GM, Bauer M, Mittman B, et al. Effectiveness-implementation Hybrid Designs: Combining Elements of Clinical Effectiveness and Implementation Research to Enhance Public Health Impact. *Medical care* 2012;50(3):217-26. doi: 10.1097/MLR.0b013e3182408812
30. Lindblad AS, Zingeser P, Sismanyazici-Navaie N. Incentives and barriers to neurological clinical research participation. *Clinical investigation* 2011;1(12):1663-68. doi: 10.4155/cli.11.153
31. Harris PA, Taylor R, Thielke R, et al. Research Electronic Data Capture (REDCap) - A metadata-driven methodology and workflow process for providing translational research informatics support. *Journal of biomedical informatics* 2009;42(2):377-81. doi: 10.1016/j.jbi.2008.08.010
32. Garber CE, Blissmer B, Deschenes MR, et al. American College of Sports Medicine position stand. Quantity and quality of exercise for developing and maintaining cardiorespiratory, musculoskeletal, and neuromotor fitness in apparently healthy adults: guidance for prescribing exercise. *Medicine and science in sports and exercise* 2011;43(7):1334-59. doi: 10.1249/MSS.0b013e318213fefb [published Online First: 2011/06/23]
33. Ferguson B. ACSM's Guidelines for Exercise Testing and Prescription 9th Ed. 2014. *The Journal of the Canadian Chiropractic Association* 2014;58(3):328-28.
34. Kietzmann JH, Hermkens K, McCarthy IP, et al. Social media? Get serious! Understanding the functional building blocks of social media. *Business Horizons* 2011;54(3):241-51. doi: https://doi.org/10.1016/j.bushor.2011.01.005
35. Ellis T, Motl RW. Physical activity behavior change in persons with neurologic disorders: overview and examples from Parkinson disease and multiple sclerosis. *Journal of neurologic physical*

For peer review only - http://bmjopen.bmj.com/site/about/guidelines.xhtml

therapy : JNPT 2013;37(2):85-90. doi: 10.1097/NPT.0b013e31829157c0 [published Online First: 2013/05/02]

36. Bandura A. Social foundations of thought and action: a social cognitive theory. Englewood Cliffs, NJ: Prentice Hall 1986.
37. Bandura A. Health promotion by social cognitive means. *Health education & behavior : the official publication of the Society for Public Health Education* 2004;31(2):143-64. doi: 10.1177/1090198104263660 [published Online First: 2004/04/20]
38. Bandura A. Self-efficacy: The exercise of control. New York, NY, US: W H Freeman/Times Books/Henry Holt & Co. 1997.
39. Maher C, Ferguson M, Vandelanotte C, et al. A Web-Based, Social Networking Physical Activity Intervention for Insufficiently Active Adults Delivered via Facebook App: Randomized Controlled Trial. *Journal of medical Internet research* 2015;17(7):e174. doi: 10.2196/jmir.4086 [published Online First: 2015/07/15]
40. Maher CA, Lewis LK, Ferrar K, et al. Are health behavior change interventions that use online social networks effective? A systematic review. *Journal of medical Internet research* 2014;16(2):e40. doi: 10.2196/jmir.2952 [published Online First: 2014/02/20]
41. Befort CA, Donnelly JE, Sullivan DK, et al. Group versus individual phone-based obesity treatment for rural women. *Eating behaviors* 2010;11(1):11-7. doi: 10.1016/j.eatbeh.2009.08.002 [published Online First: 2009/12/08]
42. Godin Leisure-Time Exercise Questionnaire. *Medicine&amp Science in Sports &amp Exercise* 1997;29(Supplement):36-38. doi: 10.1097/00005768-199706001-00009
43. Rimmer JH, Rauworth A, Wang E, et al. A randomized controlled trial to increase physical activity and reduce obesity in a predominantly African American group of women with mobility disabilities and severe obesity. *Preventive Medicine* 2009;48(5):473-79. doi: https://doi.org/10.1016/j.ypmed.2009.02.008
44. Rimmer J, Wang E, Pellegrini C, et al. Telehealth Weight Management Intervention for Adults with Physical Disabilities A Randomized Controlled Trial2013.
45. Froehlich-Grobe K, White GW. Promoting physical activity among women with mobility impairments: a randomized controlled trial to assess a home- and community-based intervention 1 1No commercial party having a direct financial interest in the results of the research supporting this article has or will confer a benefit upon the author(s) or upon any organization with which the author(s) is/are associated. *Archives of Physical Medicine and Rehabilitation* 2004;85(4):640-48. doi: https://doi.org/10.1016/j.apmr.2003.07.012
46. Froehlich-Grobe K, Lee J, Aaronson L, et al. Exercise for Everyone: A randomized controlled trial of Project Workout On Wheels in promoting exercise among wheelchair users. *Archives of physical medicine and rehabilitation* 2014;95(1):20-28. doi: 10.1016/j.apmr.2013.07.006
47. Katherine SB, John PAI, Claire M, et al. Power failure: why small sample size undermines the reliability of neuroscience. *Nature Reviews Neuroscience* 2013;14(5):365. doi: 10.1038/nrn3475
48. Bailar JC, Iii, Mosteller F. Guidelines for statistical reporting in articles for medical journals: Amplifications and explanations. *Annals of Internal Medicine* 1988;108(2):266-73. doi: 10.7326/0003-4819-108-2-266
49. Saville DJ. Multiple Comparison Procedures: The Practical Solution. *The American Statistician* 1990;44(2):174-80. doi: 10.2307/2684163
50. Saville DJ. Multiple Comparison Procedures—Cutting the Gordian Knot. *Agronomy Journal* 2015;107(2):730-35. doi: 10.2134/agronj2012.0394
51. P Burnham K, R Anderson D. Multimodel Inference: understanding AIC and BIC in Model Selection2004.

For peer review only

For peer review only - http://bmjopen.bmj.com/site/about/guidelines.xhtml

52. Burnham K AD. Model selection and multimodel inference: A practical information-theoretic approach. 2 ed: Springer-Verlag 2002.
53. Rimmer JH, Riley B, Wang E, et al. Physical activity participation among persons with disabilities: barriers and facilitators. *Am J Prev Med* 2004;26(5):419-25. doi: 10.1016/j.amepre.2004.02.002 [published Online First: 2004/05/29]
54. Salisbury SK, Choy NL, Nitz J. Shoulder pain, range of motion, and functional motor skills after acute tetraplegia. *Arch Phys Med Rehabil* 2003;84(10):1480-5. [published Online First: 2003/10/31]
55. Rimmer JH. The conspicuous absence of people with disabilities in public fitness and recreation facilities: lack of interest or lack of access? *American journal of health promotion : AJHP* 2005;19(5):327-9, ii. [published Online First: 2005/05/18]
56. Rimmer JH, Padalabalanarayanan S, Malone LA, et al. Fitness facilities still lack accessibility for people with disabilities. *Disability and Health Journal* 2017;10(2):214-21. doi: https://doi.org/10.1016/j.dhjo.2016.12.011
57. Cook KF, Molton IR, Jensen MP. Fatigue and aging with a disability. *Arch Phys Med Rehabil* 2011;92(7):1126-33. doi: 10.1016/j.apmr.2011.02.017 [published Online First: 2011/06/28]
58. Miriam W, Martin-Diener E, Bauer G, et al. Comparison of trial participants and open access users of a web-based physical activity intervention regarding adherence, attrition, and repeated participation. *Journal of medical Internet research* 2010;12(1):e3. doi: 10.2196/jmir.1361 [published Online First: 2010/02/12]
59. Jaarsma EA, Dijkstra PU, Geertzen JH, et al. Barriers to and facilitators of sports participation for people with physical disabilities: a systematic review. *Scandinavian journal of medicine & science in sports* 2014;24(6):871-81. doi: 10.1111/sms.12218 [published Online First: 2014/04/16]
60. Rimmer JH, Rubin SS, Braddock D. Barriers to exercise in African American women with physical disabilities. *Arch Phys Med Rehabil* 2000;81(2):182-8. [published Online First: 2000/02/11]
61. Rimmer JH, Silverman K, Braunschweig C, et al. Feasibility of a health promotion intervention for a group of predominantly African American women with type 2 diabetes. *The Diabetes educator* 2002;28(4):571-80. doi: 10.1177/014572170202800411 [published Online First: 2002/09/13]
62. Rimmer JH, Wang E, Smith D. Barriers associated with exercise and community access for individuals with stroke. *Journal of rehabilitation research and development* 2008;45(2):315-22. [published Online First: 2008/06/21]

For peer review only

For peer review only - http://bmjopen.bmj.com/site/about/guidelines.xhtml

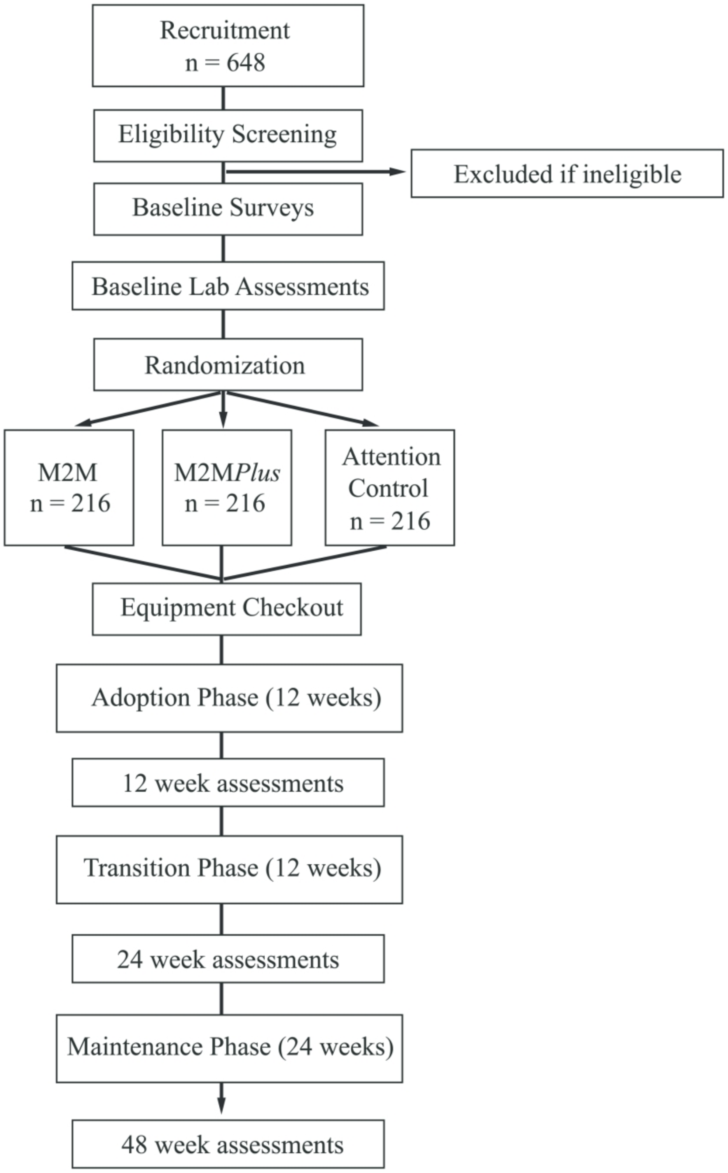

Protocol Flowchart

224x321mm (300 x 300 DPI)

TIDieR
Template for Intervention Description and Replication

The TIDieR (Template for Intervention Description and Replication) Checklist*:

Information to include when describing an intervention and the location of the information

Item number	Item	Where located **	
		Primary paper (page or appendix number)	Other † (details)
	**BRIEF NAME**		
**1.**	Provide the name or a phrase that describes the intervention.	Title Page	
	**WHY**		
**2.**	Describe any rationale, theory, or goal of the elements essential to the intervention.	1-5	
	**WHAT**		
**3.**	Materials: Describe any physical or informational materials used in the intervention, including those provided to participants or used in intervention delivery or in training of intervention providers. Provide information on where the materials can be accessed (e.g. online appendix, URL).	8-9	
**4.**	Procedures: Describe each of the procedures, activities, and/or processes used in the intervention, including any enabling or support activities.	10-11	
	**WHO PROVIDED**		
**5.**	For each category of intervention provider (e.g. psychologist, nursing assistant), describe their expertise, background and any specific training given.	11-12	
	**HOW**		
**6.**	Describe the modes of delivery (e.g. face-to-face or by some other mechanism, such as internet or telephone) of the intervention and whether it was provided individually or in a group.	10-12	
	**WHERE**		
**7.**	Describe the type(s) of location(s) where the intervention occurred, including any necessary infrastructure or relevant features.	Title, 18	

For peer review only - http://bmjopen.bmj.com/site/about/guidelines.xhtml

	**WHEN and HOW MUCH**		
**8.**	Describe the number of times the intervention was delivered and over what period of time including the number of sessions, their schedule, and their duration, intensity or dose.	9-11	
	**TAILORING**		
**9.**	If the intervention was planned to be personalised, titrated or adapted, then describe what, why, when, and how.	10	
	**MODIFICATIONS**		
**10.‡**	If the intervention was modified during the course of the study, describe the changes (what, why, when, and how).	N/A	
	**HOW WELL**		
**11.**	Planned: If intervention adherence or fidelity was assessed, describe how and by whom, and if any strategies were used to maintain or improve fidelity, describe them.	13	
**12.‡**	Actual: If intervention adherence or fidelity was assessed, describe the extent to which the intervention was delivered as planned.	N/A	

** **Authors** - use N/A if an item is not applicable for the intervention being described. **Reviewers** – use '?' if information about the element is not reported/not sufficiently reported.

† If the information is not provided in the primary paper, give details of where this information is available. This may include locations such as a published protocol or other published papers (provide citation details) or a website (provide the URL).

‡ If completing the TIDieR checklist for a protocol, these items are not relevant to the protocol and cannot be described until the study is complete.

* We strongly recommend using this checklist in conjunction with the TIDieR guide (see *BMJ* 2014;348:g1687) which contains an explanation and elaboration for each item.

* The focus of TIDieR is on reporting details of the intervention elements (and where relevant, comparison elements) of a study. Other elements and methodological features of studies are covered by other reporting statements and checklists and have not been duplicated as part of the TIDieR checklist. When a **randomised trial** is being reported, the TIDieR checklist should be used in conjunction with the CONSORT statement (see www.consort-statement.org) as an extension of **Item 5 of the CONSORT 2010 Statement.** When a **clinical trial protocol** is being reported, the TIDieR checklist should be used in conjunction with the SPIRIT statement as an extension of **Item 11 of the SPIRIT 2013 Statement** (see www.spirit-statement.org). For alternate study designs, TIDieR can be used in conjunction with the appropriate checklist for that study design (see www.equator-network.org).

TIDieR checklist

For peer review only

SPIRIT
STANDARD PROTOCOL ITEMS: RECOMMENDATIONS FOR INTERVENTIONAL TRIALS

SPIRIT 2013 Checklist: Recommended items to address in a clinical trial protocol and related documents*

Section/item	Item No	Description	Addressed on page number
**Administrative information**			
Title	1	Descriptive title identifying the study design, population, interventions, and, if applicable, trial acronym	1
Trial registration	2a	Trial identifier and registry name. If not yet registered, name of intended registry	1
	2b	All items from the World Health Organization Trial Registration Data Set	1
Protocol version	3	Date and version identifier	informed consent
Funding	4	Sources and types of financial, material, and other support	19
Roles and responsibilities	5a	Names, affiliations, and roles of protocol contributors	title page
	5b	Name and contact information for the trial sponsor	19
	5c	Role of study sponsor and funders, if any, in study design; collection, management, analysis, and interpretation of data; writing of the report; and the decision to submit the report for publication, including whether they will have ultimate authority over any of these activities	18
	5d	Composition, roles, and responsibilities of the coordinating centre, steering committee, endpoint adjudication committee, data management team, and other individuals or groups overseeing the trial, if applicable (see Item 21a for data monitoring committee)	n/a

For peer review only

For peer review only - http://bmjopen.bmj.com/site/about/guidelines.xhtml

Introduction

Background and rationale	6a	Description of research question and justification for undertaking the trial, including summary of relevant studies (published and unpublished) examining benefits and harms for each intervention	3-5
	6b	Explanation for choice of comparators	4-5
Objectives	7	Specific objectives or hypotheses	5
Trial design	8	Description of trial design including type of trial (eg, parallel group, crossover, factorial, single group), allocation ratio, and framework (eg, superiority, equivalence, noninferiority, exploratory)	5

Methods: Participants, interventions, and outcomes

Study setting	9	Description of study settings (eg, community clinic, academic hospital) and list of countries where data will be collected. Reference to where list of study sites can be obtained	5-6
Eligibility criteria	10	Inclusion and exclusion criteria for participants. If applicable, eligibility criteria for study centres and individuals who will perform the interventions (eg, surgeons, psychotherapists)	7
Interventions	11a	Interventions for each group with sufficient detail to allow replication, including how and when they will be administered	8-12
	11b	Criteria for discontinuing or modifying allocated interventions for a given trial participant (eg, drug dose change in response to harms, participant request, or improving/worsening disease)	n/a
	11c	Strategies to improve adherence to intervention protocols, and any procedures for monitoring adherence (eg, drug tablet return, laboratory tests)	13
	11d	Relevant concomitant care and interventions that are permitted or prohibited during the trial	7
Outcomes	12	Primary, secondary, and other outcomes, including the specific measurement variable (eg, systolic blood pressure), analysis metric (eg, change from baseline, final value, time to event), method of aggregation (eg, median, proportion), and time point for each outcome. Explanation of the clinical relevance of chosen efficacy and harm outcomes is strongly recommended	13-14; table 3
Participant timeline	13	Time schedule of enrolment, interventions (including any run-ins and washouts), assessments, and visits for participants. A schematic diagram is highly recommended (see Figure)	20

For peer review only - http://bmjopen.bmj.com/site/about/guidelines.xhtml

Sample size	14	Estimated number of participants needed to achieve study objectives and how it was determined, including clinical and statistical assumptions supporting any sample size calculations	14-15
Recruitment	15	Strategies for achieving adequate participant enrolment to reach target sample size	5-6

Methods: Assignment of interventions (for controlled trials)

Allocation:			
Sequence generation	16a	Method of generating the allocation sequence (eg, computer-generated random numbers), and list of any factors for stratification. To reduce predictability of a random sequence, details of any planned restriction (eg, blocking) should be provided in a separate document that is unavailable to those who enrol participants or assign interventions	12
Allocation concealment mechanism	16b	Mechanism of implementing the allocation sequence (eg, central telephone; sequentially numbered, opaque, sealed envelopes), describing any steps to conceal the sequence until interventions are assigned	11-12
Implementation	16c	Who will generate the allocation sequence, who will enrol participants, and who will assign participants to interventions	8
Blinding (masking)	17a	Who will be blinded after assignment to interventions (eg, trial participants, care providers, outcome assessors, data analysts), and how	12
	17b	If blinded, circumstances under which unblinding is permissible, and procedure for revealing a participant's allocated intervention during the trial	n/a

Methods: Data collection, management, and analysis

Data collection methods	18a	Plans for assessment and collection of outcome, baseline, and other trial data, including any related processes to promote data quality (eg, duplicate measurements, training of assessors) and a description of study instruments (eg, questionnaires, laboratory tests) along with their reliability and validity, if known. Reference to where data collection forms can be found, if not in the protocol	13-14; table 3
	18b	Plans to promote participant retention and complete follow-up, including list of any outcome data to be collected for participants who discontinue or deviate from intervention protocols	13

For peer review only - http://bmjopen.bmj.com/site/about/guidelines.xhtml

Data management	19	Plans for data entry, coding, security, and storage, including any related processes to promote data quality (eg, double data entry; range checks for data values). Reference to where details of data management procedures can be found, if not in the protocol	8
Statistical methods	20a	Statistical methods for analysing primary and secondary outcomes. Reference to where other details of the statistical analysis plan can be found, if not in the protocol	15-16
	20b	Methods for any additional analyses (eg, subgroup and adjusted analyses)	15-16
	20c	Definition of analysis population relating to protocol non-adherence (eg, as randomised analysis), and any statistical methods to handle missing data (eg, multiple imputation)	15-16
**Methods: Monitoring**			
Data monitoring	21a	Composition of data monitoring committee (DMC); summary of its role and reporting structure; statement of whether it is independent from the sponsor and competing interests; and reference to where further details about its charter can be found, if not in the protocol. Alternatively, an explanation of why a DMC is not needed	16
	21b	Description of any interim analyses and stopping guidelines, including who will have access to these interim results and make the final decision to terminate the trial	n/a
Harms	22	Plans for collecting, assessing, reporting, and managing solicited and spontaneously reported adverse events and other unintended effects of trial interventions or trial conduct	15
Auditing	23	Frequency and procedures for auditing trial conduct, if any, and whether the process will be independent from investigators and the sponsor	17
**Ethics and dissemination**			
Research ethics approval	24	Plans for seeking research ethics committee/institutional review board (REC/IRB) approval	15
Protocol amendments	25	Plans for communicating important protocol modifications (eg, changes to eligibility criteria, outcomes, analyses) to relevant parties (eg, investigators, REC/IRBs, trial participants, trial registries, journals, regulators)	15

For peer review only - http://bmjopen.bmj.com/site/about/guidelines.xhtml

Consent or assent	26a	Who will obtain informed consent or assent from potential trial participants or authorised surrogates, and how (see Item 32)	14
	26b	Additional consent provisions for collection and use of participant data and biological specimens in ancillary studies, if applicable	n/a
Confidentiality	27	How personal information about potential and enrolled participants will be collected, shared, and maintained in order to protect confidentiality before, during, and after the trial	16
Declaration of interests	28	Financial and other competing interests for principal investigators for the overall trial and each study site	19
Access to data	29	Statement of who will have access to the final trial dataset, and disclosure of contractual agreements that limit such access for investigators	16-17
Ancillary and post-trial care	30	Provisions, if any, for ancillary and post-trial care, and for compensation to those who suffer harm from trial participation	informed consent
Dissemination policy	31a	Plans for investigators and sponsor to communicate trial results to participants, healthcare professionals, the public, and other relevant groups (eg, via publication, reporting in results databases, or other data sharing arrangements), including any publication restrictions	16
	31b	Authorship eligibility guidelines and any intended use of professional writers	16
	31c	Plans, if any, for granting public access to the full protocol, participant-level dataset, and statistical code	n/a
**Appendices**			
Informed consent materials	32	Model consent form and other related documentation given to participants and authorised surrogates	appendix
Biological specimens	33	Plans for collection, laboratory evaluation, and storage of biological specimens for genetic or molecular analysis in the current trial and for future use in ancillary studies, if applicable	n/a

*It is strongly recommended that this checklist be read in conjunction with the SPIRIT 2013 Explanation & Elaboration for important clarification on the items. Amendments to the protocol should be tracked and dated. The SPIRIT checklist is copyrighted by the SPIRIT Group under the Creative Commons "Attribution-NonCommercial-NoDerivs 3.0 Unported" license.

For peer review only - http://bmjopen.bmj.com/site/about/guidelines.xhtml

BMJ Open

Rationale and Design of a Scale-Up Project Evaluating Responsiveness to Home Exercise and Lifestyle Tele-Health (SUPER-HEALTH) in People with Mobility Disabilities: A Type 1 Hybrid Design Based Effectiveness Trial

Journal:	*BMJ Open*
Manuscript ID	bmjopen-2018-023538.R2
Article Type:	Protocol
Date Submitted by the Author:	29-Nov-2018
Complete List of Authors:	Rimmer, James H. ; University of Alabama at Birmingham, UAB/Lakeshore Research Collaborative<br>Mehta, Tapan; University of Alabama at Birmingham, Department of Health Services Administration<br>Wilroy, Jereme; University of Alabama at Birmingham, UAB/Lakeshore Research Collaborative<br>Lai, Byron; University of Alabama at Birmingham, UAB/Lakeshore Research Collaborative<br>Young, Hui-Ju; University of Alabama at Birmingham, UAB/Lakeshore Research Collaborative<br>Kim, Yumi; University of Alabama at Birmingham, UAB/Lakeshore Research Collaborative<br>Pekmezi, D; University of Alabama at Birmingham, Department of Health Behavior<br>Thirumalai, Mohanraj; University of Alabama at Birmingham, Department of Health Services Administration
**Primary Subject Heading**:	Rehabilitation medicine
Secondary Subject Heading:	Research methods
Keywords:	telerehabilitation, mobility disabilities, telehealth, exercise, disability, mhealth

SCHOLARONE™
Manuscripts

Rationale and design of a Scale-Up Project Evaluating Responsiveness to Home Exercise and Lifestyle Tele-Health (SUPER-HEALTH) in people with mobility disabilities: A type 1 hybrid design based effectiveness trial

James Rimmer,[1] PhD, Tapan Mehta,[1] PhD, Jereme Wilroy,[1] PhD, Byron Lai,[1] PhD, Hui-Ju Young,[1] PhD, Yumi Kim,[1] MA, Dorothy Pekmezi,[1] PhD, Mohanraj Thirumalai,[1] PhD

Author affiliations

[1]University of Alabama at Birmingham, Birmingham, Alabama

Author Contributions

JR provided content for the introduction and discussion. TM provided content for the data analysis and power calculation sections. MT provided all content on app development and social networking section. JW compiled the first full draft of the manuscript, providing content for recruitment section, and made all revisions provided by coauthors. BL provided all content on equipment and technology. HY provided all content for the exercise video section. YK provided content on data management protocol. DP provided content for theoretical framework section. All authors reviewed paper in full and provided revisions on the entire paper.

Keywords: eHealth, Physical Activity, Physically Disabled, mHealth, Humans

Word count: 6,137

Corresponding Author:

Mohanraj Thirumalai

1720 2nd Avenue South

Birmingham, AL 35294-0113

mohanraj@uab.edu

205-934-7189

ABSTRACT

Introduction: Rates of physical inactivity among people with physical disabilities are substantially higher than in the general population while access to home-based exercise programs tailored are almost non-existent. Using a theory-driven eHealth platform, an

For peer review only - http://bmjopen.bmj.com/site/about/guidelines.xhtml

For peer review only

innovative exercise program referred to as movement-to-music (M2M) will be delivered as a customized, home-based exercise intervention for adults with mobility disabilities.

Methods and analysis: Participants are being recruited for this type 1 hybrid design based effectiveness trial through outpatient clinics at a large rehabilitation center and randomized to one of three groups: a) M2M; b) M2M plus social networking (M2M^{plus}); and c) attention control (AC). The intervention includes a 12-week adoption phase, 12-week transition phase, and 24-week maintenance phase, at which the collection of objective measures on exercise, fitness and self-reported measures on health will be obtained at the start of each phase and at follow-up. The study compares the effectiveness of M2M and M2M^{plus} in increasing physical activity (primary outcome), adherence, fitness, and physical functioning compared to the AC group and examine the mediators and moderators of the treatment effect.

Ethics and dissemination: The Institutional Review Board of The University of Alabama at Birmingham granted full approval: (IRB-160923002). Dissemination of findings will include publication in peer-reviewed journals, presentations at regional, national, and/or international meetings, and the National Center on Health, Physical Activity and Disability (NCHPAD, www.nchpad.org). This study will strengthen our understanding of the potential benefits of eHealth exercise interventions for people with physical disabilities and build upon strategies that aim to recruit larger samples in exercise trials.

Trial registration number: NCT03024320

Strengths and limitations of this study

- The trial is the largest of its kind exploring the use of telehealth technology to improve access to regular and sustainable home exercise among an underserved population of adults with mobility disabilities.

For peer review only

For peer review only - http://bmjopen.bmj.com/site/about/guidelines.xhtml

- The trial addresses a growing need among physicians treating patients with mobility disabilities to have easily accessible e-Health home exercise videos that can be tailored to the functional level of their patients.
- Utilizing constant monitoring technology will allow researchers to know when participants are not engaged in the intervention and prompt coaching calls to reduce dropout.
- Recruitment will occur through a single rehabilitation hospital network and may not be generalizable to other regions across the United States and other countries.

For peer review only

BACKGROUND

Despite what is known about the positive effects of exercise in improving health and function,[1] people with physical disabilities remain one of the least active and obese[2-8] populations in society. In the latest data from the Centers for Disease Control and Prevention, nearly one half of all adults with disabilities get no aerobic physical activity.[9] These patterns of low physical activity become more problematic across the lifespan when the effects of the natural aging process are compounded by years of sedentary living, poor nutrition and severe deconditioning.[10-13]

In addition to the physical impairments associated with a disability limiting physical activity, initiating and maintaining a physically active lifestyle is difficult for people with physical disabilities due to a number of barriers faced in the home, built environment (neighborhood, community-based exercise and recreation facilities) and healthcare system.[14] Dozens of papers have reported that the built environment creates substantial limitations in accessing outdoor and indoor physical activity programs and venues.[15-18] Outdoor exercise may be challenging or impossible because many neighborhoods either lack sidewalks or have badly damaged surfaces presenting a high risk of falling among people who have poor balance and/or use wheelchairs.[19] Also, high traffic volume can be intimidating for many people with a mobility disability who have difficulty getting across streets in the time allotted by the traffic light.[20] In terms of getting to an indoor facility such as a fitness center, lack of accessible transportation is often the number one barrier to using these facilities followed by cost of the membership.[20]

Recent data found that people with physical disabilities who were advised by their doctor to exercise were 82 percent more likely to do so.[9] However, the current healthcare system continues to provide little guidance to people with physical disabilities on how to exercise, leaving many, if not most, minimally prepared to manage or improve their health and fitness.[21 22] Therefore, healthcare providers require exercise programs that they can recommend to their

For peer review only

For peer review only - http://bmjopen.bmj.com/site/about/guidelines.xhtml

patients with disabilities that are evidence-based, easily accessible, and tailored to the specific functional and health-related needs of the individual.

In recent years, Internet-based interventions targeting health issues such as nutrition, smoking, physical activity, or multiple health behaviors have become increasingly popular.[23] These interventions have several advantages for people with physical disabilities. In addition to the interactive and convenient nature of Internet-based programs, health professionals can utilize Web-platforms to provide tailored information to individual users and potentially reach large groups of people with physical disabilities at relatively low cost. A notable benefit of eHealth programs for people with physical disabilities includes the ability to participate at home, which is the most convenient place for many people with physical/mobility disabilities to engage in sustained physical activity behavior. If designed properly, home-based exercise allows for prompt feedback, individual tailoring, and continued personalized guidance and social support.[24] Thus, Internet-based exercise programs create an accessible venue for people with physical disabilities to achieve the exercise regimens prescribed by a health professional and can potentially include individuals who do not have access to conventional community-based exercise programs.

To fully address the barriers to accessing exercise, including the need for healthcare providers to have off-the-shelf resources that they can quickly provide to their patients, technology can create exercise opportunities in the comfort of a person's home to enhance long-term participation.[25 26] This is an avenue that is currently not available in most communities across the U.S. Therefore, the proposed scale up exercise intervention will enroll a large cohort of adults with mobility disabilities from a university-based outpatient rehabilitation center, to determine if an innovative technology-based exercise program at the home can achieve improvements in physical activity adherence, health and functional outcomes. The study is referred to as SUPER-HEALTH, which stands for *S*cale-*U*p *P*roject *E*valuating *R*esponsiveness to *H*ome *E*xercise *A*nd *L*ifestyle *T*ele-*H*ealth.

For peer review only - http://bmjopen.bmj.com/site/about/guidelines.xhtml

This primary aim of this study is to test the effectiveness of a home-based e-Health exercise program for increasing physical activity among a clinical population of people with mobility disabilities. The intervention, referred to as Movement-to-Music (M2M), is being compared to an enhanced version of M2M that includes social networking (M2M^{plus}). Both of these interventions are compared to an attention control (AC) group. Secondary aims include those related to intervention effectiveness and implementation. Regarding effectiveness, aims include (1) estimating improvements in health (pain, sleep, quality of life) and physical function (balance, strength, endurance) between M2M and M2M^{plus} groups compared to the AC group; and (2) assessing mediators and moderators of social cognitive theory (self-efficacy, self-regulation, social support, outcome expectancies) and demographic factors (age, race, disability type) of the hypothesized treatment effect to understand for whom and how the intervention is effective. Implementation is assessed via participant uptake of the intervention, whereby the aim is to explore participant flow throughout all stages of the study (contact through enrollment and intervention adoption through intervention maintenance).

METHODS

This protocol was approved by the University's Institutional Review Board (IRB-160923002) and is registered with ClinicialTrials.gov (#NCT03024320) as a phase III clinical trial. This paper follows the SPIRIT checklist[27], TIDieR checklist[28], and conduct and reporting of the trial follows CONSORT guidelines.[29]

Study design

SUPER-HEALTH is a single site, three-arm parallel group type 1 hybrid design based effectiveness trial[30 31] evaluating the effects of two intervention groups (M2M and M2M^{plus}) compared to AC in people with mobility disabilities, with assessments conducted at four time points: baseline, 12 weeks, 24 weeks, and 48 weeks.

Recruitment

For peer review only - http://bmjopen.bmj.com/site/about/guidelines.xhtml

Participants are being recruited from outpatient clinics of a large university-based rehabilitation center through physicians and their staff and by physical mail-outs from January 2018 until the desired sample size is obtained. Supplementary recruitment strategies include brochures placed in clinics, society events, newsletters, advertisements, and word of mouth. Additionally, recruitment will include screening more male participants than females.

In order to enhance the likelihood of enrollment through clinician referral,[32] research staff attend periodic meetings with the physicians in the outpatient center. Recruitment from outpatient clinics include: 1) direct referrals from physicians and their staff; 2) indirect referrals through advertisement materials in waiting room; 3) targeted in-person visits by research staff on the day of a patient's clinic appointment; and 4) routine recruitment visits to the waiting room by research staff. Physicians have been instructed to hand out brochures that include the study objectives and contact information. The identification of eligible participants for in-person visits occurs through the review of medical records and clinic appointments using the password-protected hospital database. After potential participants are identified, the recruitment coordinator greets the individuals in the waiting room, provides an overview of the study objectives, and consents individuals interested in participating in the study.

Physical mail-outs are being delivered to potential participants that are identified from the NIH-funded National Center for Biomedical Computing based at Partners HealthCare System: Informatics for Integrating Biology and the Bedside (i2b2) [https://www.i2b2.org/about/index.html]. The i2b2 has been used to identify study cohorts within the outpatient rehabilitation clinics and address research questions by integrating a wide variety of clinical data sources. After an initial search query of participants located within the same state where the study is being conducted, and who attend one of the outpatient clinics, we identified approximately 5,400 people with neuromuscular disorders and musculoskeletal conditions. This list directs the physical mail-outs, which include a flyer that describes the study and a letter co-signed by the chair of department overseeing the clinics and the principal investigator. Mail-outs

For peer review only - http://bmjopen.bmj.com/site/about/guidelines.xhtml

are being sent in batches of 100 every 2 to 4 weeks and will be increased or decreased depending on the response rate.

Exclusion criteria

Individuals with a mobility disability are eligible for the study. In order to remain consistent with other studies,[4] this was defined as self-reported difficulty (1) walking (some, much, unable to do) without special equipment use; (2) walking one-quarter of a mile; (3) remaining on feet for more than two hours; (4) taking 10 uninterrupted steps; (5) kneeling, stooping, or crouching; or (5) standing up from an armless straight chair. Individuals who meet the following criteria are not eligible to participate:

- accumulating more than 60 minutes of moderate/vigorous physical activity per week;
- do not report having a diagnosis of a physical/mobility disability;
- not within working age (18 to 64 yrs. of age);
- currently enrolled in a structured exercise program over the past 6 months;
- unable to use upper, lower or both sets of extremities to exercise;
- unable to converse and read English;
- medically unstable to perform home exercise as determined by their physician;
- cognitive impairment that may preclude self-directed daily activities
- no Wi-FI Internet access.

Study Procedures

The study flow diagram includes three phases of the intervention, Adoption (weeks 1 to 12), Transition (weeks 13 to 24), and Maintenance (weeks 25 to 48) (Figure 1). This phased approach allows for gradual adjustment in the dosage of the intervention and the ability to capture changes within and across phases. Details of the interventions offered in each arm are shown in Table 1.

[Insert Figure 1]

[Insert Table 1]

For peer review only - http://bmjopen.bmj.com/site/about/guidelines.xhtml

All data storage is established via the Research Electronic Data Capture (REDCap),[33] an electronic data capture system. When potential participants fill in their information via the study website, it is automatically stored in REDCap. Potential participants' information with other methods of contact (e.g., mail-back, call) is manually entered into REDCap by the recruitment coordinator. The recruitment coordinator reaches out to potential participants within 48-72 hours based on their preferred contact methods (i.e., phone, emails) for more information regarding the study as well as participation eligibility. If eligible, the participant will be consented over the phone and will then receive baseline surveys to be completed online prior to testing visit. Once participants complete the baseline testing they will be randomized.

After the group allocation, participants receive a study designated email address which is uploaded to REDCap. Surveys are automatically sent at the remaining follow-up time points, 12 weeks, 24 weeks, and 48 weeks, to the new email address. There is a 10-day window open before and after the exact date of follow-up data collection based on the initial lab testing date. Participants receive the same email a maximum of five times (1 per day) if they do not complete the survey. When a research staff member notices the missing data after 5 days, the participant is contacted by phone.

Fitness and physical function measures are assessed in a research lab within a state-of-the-art universally designed community health and fitness facility. Transportation is provided to the participants, if needed. After a review of the consent form, the lab staff completes anthropometric measures (height, weight, waist circumference) and vital signs (blood pressure, heart rate). Participant then complete a battery of fitness and physical function measures (see Table 2) while lab staff record results in a data collection packet.

Interventions

For peer review only - http://bmjopen.bmj.com/site/about/guidelines.xhtml

After the lab assessments are completed, the technology coordinator provides the participant with the appropriate equipment based on their group assignment and functional level. The equipment includes a tablet (ASUS Zenpad 3s 10), a tablet case (Fintie Folio Stand Cover), stylus, Fitbit Charge 2 HR to monitor physical activity, a set of two pound wrist weights (SPRI Thumblock Wrist Weight) and an optional stand (Musician's Gear Folding Music Stand). The technology coordinator then familiarizes participants with the equipment and also guides participants through the tablet app. Equipment instructions cover basic operation (e.g., turning the devices on and off, connecting to Wi-Fi, etc.) and maintenance skills (e.g., cleaning the devices and damage prevention). Regarding app use, participants are carefully guided through the tablet app content and functions. Following these instructions, participants are then asked to independently navigate through the app and view all content.

The tablet app includes the foundational elements required for rapid deployment of the video and textual content, and is password protected to enable detailed usage tracking. The home page of the app provides brief weekly objectives and links to newly received content. Other features of the app include a video and article library, calendar for scheduling, and activity dashboard with Fitbit data. In addition to these features, the M2M^{plus} group's version of the app includes elements that support social interaction. These ancillary features include the ability to private-message other users, leaderboards that display user progress, a user profile, newsfeed, and the ability to comment on and "like" the exercise videos. The app version for the intervention groups (M2M and M2M^{plus}) includes exercise video content, while the AC group will only receive access to the infographics and the Fitbit dashboard.

Educational Resources

Each group receives textual content (i.e., articles, infographics) aimed at improving health through lifestyle changes and tailored to adults with mobility disabilities. This content includes physical activity recommendations, how to maintain activity, developing healthy nutrition habits, ways to reduce stress, and other health-related information. Each group receives this content

For peer review only - http://bmjopen.bmj.com/site/about/guidelines.xhtml

weekly for 12 weeks (Adoption phase), bi-weekly for the following 12 weeks (Transition phase), and monthly for the last 24 weeks (Maintenance phase). Exercise Video Content

Exercise videos include movements that were adapted from an onsite program that has been conducted at a state-of-the-art fitness facility for people with disabilities. The onsite program incorporated an extensive set of movement routines that were choreographed to the functional needs of people with a range of physical disabilities. For the present study, we modified the onsite program into a video-based package that could be performed independently in the home. Program modifications were made using a framework referred to as MAPPETT (*M*odality, *A*daptation, *P*osition, *P*attern, *E*quipment, *T*empo, and *T*ime) to systematically document exercise adaptations. The *M*odality (range of motion/flexibility, muscle strength, cardiorespiratory fitness, and balance), *T*empo (a given range of music beat per minute), and *T*ime (duration of each exercise routine) are intervention elements that are structured and need to be delivered accordingly. The *P*osition (i.e., standing or sitting), *P*attern (i.e., anatomical planes, types of motion, static/dynamic, movement sequence), and *E*quipment (i.e., wrist weight, ankle weight, chair) are elements that can be adapted (*A*daptation) to people with different functional capacities. The goal of the home-based program is to slowly and safely progress participants towards achieving and maintaining 150 minutes of moderate-intensity physical activity per week, which is based on the guidelines provided by the American College of Sports Medicine.[34 35]

The exercise intervention is referred to as M2M. The M2M intervention includes three broad sub-programs or levels that can be arranged by program staff to meet the functional needs of the participant: 1) able to use all four limbs; 2) able to use only the upper limbs (e.g., someone with lower extremity paralysis); and 3) able to use only one side of the body to exercise (e.g., individuals with stroke/hemiparesis). The determination of the level for each participant is based on their baseline physical function assessments. Each M2M session includes four sets of exercise routines: flexibility, muscle strength, cardiorespiratory fitness, and

For peer review only - http://bmjopen.bmj.com/site/about/guidelines.xhtml

balance. The video ends with a cool down breathing routine. Participants are instructed to complete the assigned M2M exercise videos three times per week. The session duration and exercise intensity is gradually increased throughout the program.

The exercise videos are delivered across the three intervention phases: 1) Adoption, weeks 1-12; 2) Transition, weeks 13-24; and 3) Maintenance, weeks 25-48. During the Adoption phase, one new video is uploaded each week to the eHealth platform. The exercise program begins with a total of 15 minutes of exercise at week 1 and slowly progresses to 90 minutes at week 12. The rationale for starting the program at a low duration is to help participants build confidence and gradually incorporate the exercise program into their daily life. Each week the videos include newly added routines that are guided by an M2M instructor and an actor with a disability that matches the functional level of the participants. When a new routine is introduced, the first segment of the video shows the M2M instructor breaking down the routine and explaining each movement pattern (guided exercise portion). In the following week, the guided exercise portion of the same routine is removed from the video so that the participants can perform the routine directly with music. Participants have the ability to view previously completed exercise videos in their video library.

During the Transition and Maintenance phases, one new video is added biweekly (twice a month) and monthly, respectively. The goal of the Transition phase is to progress participants to a goal of 150 minutes of moderate-intensity exercise.[34 35] The Transition phase starts with three 30-minute sessions at week 13 of the intervention (week 1 of this phase) and ends with three 50-minute sessions at week 24 (150 minutes/week). For the Maintenance phase, the session duration remains the same and there are new monthly videos added as an incentive to encourage participants to adhere to the study protocol by using new M2M routines.

Social Networking System

A Social Networking System (SNS) is integrated into the eHealth platform for the M2M^{plus} group. The SNS contains the essential building blocks of social media including (1) identity, (2)

For peer review only - http://bmjopen.bmj.com/site/about/guidelines.xhtml

sharing, (3) presence, (4) relationships, (5) reputations, (6) groups, and (7) conversations.[36] Additionally, M2M^{plus} participants join a weekly video conference group discussion session led by the project coordinator who has expertise in motivational interviewing strategies. The purposes of these sessions are to stimulate discussion among participants and to encourage them to support each other. Each session incorporates behavioral change strategies that have been modified from previous research with adults with physical disabilities.[37] The sessions focus on topics related to constructs from the Social Cognitive Theory (the theoretical background for this study): social support, overcoming barriers, and preventing relapse.

Theoretical Framework

The intervention is grounded in the Social Cognitive Theory,[38] which provides a comprehensive, multi-level model of behavioral change emphasizing personal and environmental factors that lead to the adoption and/or maintenance of health promoting behavior.[39] According to the Social Cognitive Theory, health behavior change is based on reciprocal determinism of the interplay of 3 domains: (1) the behavior, which involves performance or mastery by the individual (behavioral capability, self-regulation); (2) personal factors that involve cognitive, affective and biological events (e.g., self-efficacy); and (3) the environmental factors that facilitate or impede change (social and physical environmental factors).[39] Thus, the intervention targets exercise self-efficacy through self-regulation strategies (incremental goal setting, reinforcements using infographics (all groups) and by including adults with mobility disabilities as demonstrators for exercise videos (M2M and M2M^{plus} only). These approaches focus on mastery experiences, social modeling and verbal persuasion and thus are well aligned with Bandura's research on the key influences on self-efficacy.[40] Social support for physical activity intervention studies among individuals without physical disabilities, such as social networking (e.g., messages, posts, newsfeeds)[41 42] and group discussion sessions[43] led by research staff (M2M^{plus} only).

Attention control group

For peer review only - http://bmjopen.bmj.com/site/about/guidelines.xhtml

In addition to the Fitbit, the AC group receives a tablet that only includes the educational materials. After the 48-week assessment is completed, participants receive access to the exercise videos.

Randomization

Eligible participants are randomized into one of three arms with 1:1:1 allocation ratio using a permuted block randomization approach where the block size is unknown to the intervention staff. The randomization sequence is generated a priori using a computer-generated random schedule in a permuted block (SAS version 9.4). The randomization schedule is then embedded into a randomization module in REDCap.[33] This system allows researchers to manage the information with a higher level of security, remove physical envelop, and set individual level of blinding within the system.

Staff performing recruitment, outcome measurements, and data entry for the primary outcomes are blinded to group allocation. However, due to the nature of the intervention, it is not possible to blind the staff administering the interventions or the participants. Participants are instructed not to inform the data collection staff of their intervention status when they return for follow-up measures.

Intervention Fidelity

The intervention uses the latest eHealth technology as a means to deliver and monitor the intervention. Each participant receives the latest in activity monitoring (Fitbit), streaming, content (M2M and M2M^{plus} only), and a social networking platform (M2M^{plus} only), which paired with the use of cloud-based technology allows for the intervention to be administered in the home. Our intervention fidelity plan (Table 2) is based on the *Best Practices and Recommendations from the NIH Behavior Change Consortium (BCC).* The BCC recommendations are intended to link theory and application across five primary study phases, including study design, provider training, monitoring and improving the delivery of the intervention, and monitoring and improving the enactment of intervention skills.

For peer review only - http://bmjopen.bmj.com/site/about/guidelines.xhtml

[Insert Table 2]

Outcome measures

Lab-based research outcome measures are administered at baseline, 12 weeks, and 48 weeks by independent evaluators blind to treatment assignment. Survey measures are automatically delivered by the REDCap system at baseline, 12 weeks, 24 weeks, and 48 weeks.

The primary outcome is change in rates of exercise participation, measured via self-report by the Godin Leisure-Time Exercise Questionnaire (GLTEQ),[44] at the four time points. Secondary effectiveness outcomes include health and physical function measures, which involve a battery of tests performed at baseline, 12 weeks, and 48 weeks (see Table 3). In addition, anthropometric measures and vital signs are assessed at each testing visit. Short forms developed by the National Institutes of Health (NIH) Patient-Reported Outcome Measurement Information System (PROMIS) are used to measure symptoms and quality of life indicators. The PROMIS short forms used for this study include sleep disturbance, pain, depression, anxiety, physical function, and social interactions. Social cognitive theory measures include surveys and scales on exercise goal-setting, barriers to physical activity, outcome expectations for exercise, social support, and exercise self-efficacy. Other measures include app usability, eHealth literacy assessment, and demographics (i.e., race, sex, age). A brief tabulation of the measures for this study including information on key outcome variables, covariates, and time points is shown in Table 3.

[Insert Table 3]

Outcomes related to participant uptake include quantitative measures of participant flow throughout the intervention. Data for participant flow includes the number of individuals recruited, screened, and enrolled, along with intervention adherence (weeks 1-24) and maintenance data (weeks 25-48). Within these stages, the number of participants that withdraw and the reasons for withdrawal are recorded. Adherence is defined as the percentage the participant meets or exceeds the study weekly exercise protocol, which is measured by how

For peer review only - http://bmjopen.bmj.com/site/about/guidelines.xhtml

many minutes the individual views the exercise videos divided by the weekly prescription. The week 1 video begins with 5 minutes of exercise, and each participant is asked to complete the videos three times each week equaling a total of 15 minutes for the first week. This increases approximately 5 minutes each week until reaching 150 minutes of exercise video viewing per week (50 minutes of exercise completed three times) by the end of the transition phase (week 24). Maintenance is defined as the percentage the participant continues to meet the weekly exercise protocol prescribed from weeks 25-48, and the protocol will remain at 150 minutes per week. To improve adherence and ensure delivery of the intervention, the project coordinator monitors weekly data uploaded from the Fitbit and tablet that include the date, total steps per day, and a log for the exercise videos, which includes a timestamp, title of video, and the number of minutes spent viewing. If there is no Fitbit data for more than seven days or if the amount of video minutes completed in a given week is below 25% of those prescribed, the participant is contacted by research staff trained in motivational interviewing techniques. The purpose of the call is to gather information on non-adherence and aid the participant in creating an action plan for meeting the prescribed activity. Adherence across groups will be compared via: 1) the percentage of days that individuals use the Fitbit compared to those prescribed (number of times worn divided by total sessions), and 2) attrition (number of individuals that withdraw from the trial).

Power and sample size

A sample size of 648 participants resulted from conducting conservative sample size calculations with minimal assumptions, which involved the following assumptions: 80% power, two sided t-test comparing changes in the primary outcome of physical activity, family-wise error rate of 0.05 to account for multiple testing, attrition rate of 36%, and intent to treat analyses. Each pairwise comparison for the primary outcome (M2M versus AC, M2Mplus versus AC and M2M versus M2Mplus) is considered as a family of hypotheses. We did not account for multiple comparisons (between different arms) since these are planned a priori. A sample size of 648,

For peer review only - http://bmjopen.bmj.com/site/about/guidelines.xhtml

which result in 415 participants as completers after accounting for the 36% attrition, provides 90% power to detect an effect size of 0.32 (Cohen's d) between any of the pairwise comparisons aforementioned above.

We can consider the 2 sets of secondary outcomes (4 objective measures and 4 patient reported measures – see Table 3) as separate families of hypotheses. Again, we do not account for multiple comparisons (pairwise comparison between 3 arms) but do account for multiple outcomes testing within each pairwise comparison. Hence, assuming a type 1 error rate of 0.0125 with a conservative Bonferroni correction for multiple testing, we will have 90% power to detect an effect size of 0.373.

Previous effect sizes (ES) estimated for changes in exercise from two web-based interventions reported ES of 0.6129 and 0.8, respectively,[45 46], and ES achieved from two home-based behavioral interventions to increase exercise in wheelchair users above 0.5.[47 48] Our minimum detectable effect size of 0.375 is much smaller than the aforementioned effect sizes to account for the variability in the effect sizes and winner's curse[49] (anticipated scenario given that we aim to recruit a far larger sample size). An assumed attrition rate (AR) of 36% was based on the study by Froehlich-Grobe et al.[48] While our prior studies had an AR as low as 13%, the eHealth literature cautions of higher attrition rates. Achieving lower ARs may possibly relate to the role of health/motivational coaches in the study. Finally, we will be able to bring more efficiency in terms of power and effect size since our primary statistical analysis is based on mixed modeling approach. The mixed modeling approach accounts for correlation between measurements from the same participant that improves power for the same effect size or provides us the ability to detect a lower effect size for the same power and aforementioned assumptions.[49] Mixed modeling technique also utilizes all the data available (that is imputes any missing outcome data). Hence, despite attrition we will have a larger sample size while conducting longitudinal analyses, which would again bring more power/sample efficiency.

For peer review only - http://bmjopen.bmj.com/site/about/guidelines.xhtml

Analyses

All statistical analyses are conducted in an intent-to-treat manner, at the individual level. For the single primary outcome statistical significance will be evaluated at a 2-tailed hypothesis-wise error rate of 0.05. Consistent with published guidelines for statistical reporting, exact p-values (rather than, e.g., "$p < 0.05$" or "NS") will be reported.[50] This allows readers who may have their own opinions about multiple comparison corrections to know the raw uncorrected result and make their own judgments about statistical significance especially given that the significance tests conducted for primary outcome are specified a priori and modest in number. As Saville[51 52] noted, in multiple comparison issues, there is no right answer and each investigator must ultimately "cut the Gordian knot" themselves. For the secondary outcomes statistical significance is evaluated at a family-wise error rate of 0.05 after accounting for multiple outcomes testing. Based on the p-values for all analyses reported, we will also report the false discovery rate and false discovery proportion.

In general, missingness in outcomes is handled by mixed models for repeated measures data. Quality control (QC) includes descriptive and graphical approaches to summarize baseline characteristics of all key variables.

For the primary outcome of physical activity, we will compare two planned pairwise comparisons. The primary aim includes two hypotheses stating that the M2M and M2M^{plus} interventions will lead to greater increases in exercise from baseline to the transition phase when compared to the AC group. The outcome variable will be a time-varying measure (two time points: baseline and transition phase) of exercise. Using self-report data from the GLTEQ, a Health Contribution Score will be calculated.[44] The baseline exercise measure will be calculated as the average exercise week across a 7-day period prior to starting the intervention (Adoption phase). Similarly, the average exercise for a 7-day period at the end of each intervention phase will be estimated. The main hypothesis addresses the change in activity between baseline and transition phases across intervention groups. Baseline covariate

For peer review only - http://bmjopen.bmj.com/site/about/guidelines.xhtml

measures will be included if differences are identified across intervention arms, or if inclusion of these covariates improves the corrected Akaike Information Criterion (AIC).[53 54]

In addition to any baseline covariates, fixed effects will include intervention arms (M2M, M2M[plus]), AC, Time (baseline, transition) and an Arms X Time interaction. Contrast statements will be used to test the null hypotheses in conjunction with the fitted model coefficients. The estimates of the change in exercise for each arm and their 95% confidence interval will also be reported. Secondary data analyses will include testing for non-linearity in the differences between change scores in exercise across the intervention phases and testing whether there were greater increases in exercise in M2M and/or M2M[plus] compared to AC from baseline to end of adoption and baseline to transition.

Ethics and dissemination

The IRB will remain informed of any protocol changes or adverse events for the safety of participants. The recruitment and project coordinators will consent each participant to the study. Once the participant signs the informed consent, they receive a unique research ID code and no personally identifiable information will be linked to data collected for this study. Quality assurance procedures are used to minimize missing data, possible errors, and to correct errors before final database lock. After completion of data entry, two additional research staff (project coordinator and data reviewer) audit data periodically and lock the record. When the survey is submitted to REDCap, research staff receive a notification via email and review the data within 48 hours. An independent Data Safety and Monitoring Committee is not necessary due to the minimal risk associated with participant outcomes. All data collection will be overseen by the principal investigator and co-investigators, including a biostatistician and physician.

Findings from this study will be shared publicly and disseminated by: (1) publication in peer-reviewed journals; and/or (2) presentations at regional, national, and/or international meetings. Findings will also be disseminated through the National Center on Health, Physical Activity and Disability (NCHPAD, www.nchpad.org), which has over 20,000 email subscribers. If

For peer review only - http://bmjopen.bmj.com/site/about/guidelines.xhtml

the M2M exercise intervention achieves successful outcomes, we will make the program available to other medical centers through the NCHPAD website. We are committed to the sharing of final research data, being mindful that the rights and privacy of our research participants must be protected at all times, that there is the need to protect patentable and other proprietary data (ie, our web-based platform), and that restrictions on data sharing may be imposed by agreements with third parties. Published data with non-identifiers will also be shared with the 2018 Physical Activity Guidelines committee upon request.

Patient and Public Involvement

There was no patient or public involvement in the design of this study.

DISCUSSION

This paper has presented the background and design for a randomized controlled trail investigating the effectiveness of a home-based, eHealth exercise program for adults with mobility disabilities. To our knowledge, this study is the largest exercise trial ever conducted on people with mobility disabilities.

Engaging in the recommended levels of physical activity (150 minutes of moderate intensity physical activity per week) can be extremely challenging for many people with mobility disabilities, who have limited to no access to home exercise equipment, and indoor and outdoor physical activity programs and venues (e.g., outdoor parks, fitness facilities).[15-18 55] In particular, outdoor exercise, used by many people who do not have a mobility disability (e.g., walking, jogging, hiking, cycling), may be difficult or impossible to perform by people with mobility disabilities. This is because neighborhoods either lack sidewalks or have surfaces that are badly damaged imposing a risk of falls; high traffic volume makes it cumbersome to get across streets; hilly terrain may be too difficult to traverse; and for people who use wheelchairs, manually pushing a chair for an extended period of time to achieve the recommended levels of exercise may induce or worsen shoulder pain.[56] Indoor exercise at a local fitness center has also been documented as being largely inaccessible to people with mobility disabilities due to

For peer review only - http://bmjopen.bmj.com/site/about/guidelines.xhtml

lack of accessible exercise equipment and programs and [57 58] transportation. Membership costs often exceed economic resources.[55]

The low level of exercise participation observed in people with mobility disabilities likely have a negative impact on their health. Nearly one-half of people with disabilities are physically inactive, which the U.S. Centers for Disease Control and Prevention defines as not engaging in *any* aerobic physical activity, and this large inactive subgroup is 50 percent more likely to have a chronic disease compared to those who get the recommended amount of aerobic physical activity on a weekly basis.[9] Unfortunately, the current U.S. healthcare system is woefully underprepared to prescribe recommended exercise to people with mobility disabilities, leaving many, if not most, minimally prepared to manage or improve their own health and fitness as they grow older and have more health issues.[21 22 59] Health care providers clearly need more exercise resources that they can recommend to their patients with disabilities that are evidence-based, easily accessible, and tailored to their specific functional level.

The SUPER-HEALTH project addresses this growing epidemic of physical inactivity among people with mobility disabilities by providing simple access to an exercise program tailored to the individuals' functional level and performed in the comfort of their home. Delivery of the exercise program through a tablet app offers opportunities to (a) reach isolated populations who have no other way to obtain regular exercise tailored to their unique needs; (b) eliminate transportation difficulties needing to get to a facility-based intervention; and (c) allow the individual to exercise at a time of the day that is convenient for them. Other benefits include their low cost (compared to on-site programs) and the ability for individuals to work at a self-selected pace in a comfortable environment.[24 60]

There are several novel features embedded into SUPER-HEALTH. First, eHealth (telehealth) technology's capacity to provide patient engagement related to health/wellness can create a connected healthcare model that links individuals to trained health/exercise professionals and their healthcare provider offering them greater participation in their own care.

For peer review only - http://bmjopen.bmj.com/site/about/guidelines.xhtml

Second, with transportation and program costs being two of the most common barriers reported among people with mobility disabilities in terms of obtaining regular exercise,[17 61-64] home-based programs with cloud-based technology and social support hold strong potential for reaching them in their home. Third, the telehealth technology used in this study is complemented by the latest innovation in activity monitoring. The Fitbit is a state-of-the-art accelerometer that provides an objective measure of physical activity allowing the participant to receive activity monitoring throughout the program . Fourth, the Internet-based home training package (aerobics, strength, flexibility and balance) is provided in the form of music, which offers greater levels of enjoyment and increases its potential for future scalability. The exercise routines can be mixed and matched with new music and movement patterns, which keeps the exercise routines novel. Finally, the use of an eHealth platform has the potential to shift relatively large segments of the population of people with mobility disabilities from inactive to physically active in a sustainable way that extends beyond the intervention timeframe.

CONCLUSION

SUPER-HEALTH is a multi-level scale up exercise eHealth intervention using healthcare providers as the point-of-entry for enrolling patients with mobility disabilities into the study. If SUPER-HEALTH proves efficacious, promotion of health-enhancing exercise for people with mobility disabilities through an eHealth movement-to-music (M2M) intervention (targeted to individual functional level) has strong potential to reach large numbers of individuals who are currently not being prescribed a home-based exercise program by their healthcare provider.

Study sponsorship, funding and organization

This work is supported by a grant from the National Institutes of Health, Eunice Kennedy Shriver National Institute of Child Health and Human Development (1R01HD085186). This study is sponsored by the University of Alabama at Birmingham and will be coordinated by the Lakeshore Foundation, Birmingham, Alabama. The study funder will have no role in the conduct

For peer review only - http://bmjopen.bmj.com/site/about/guidelines.xhtml

or evaluation of the trial, nor the authority over the study design, analysis, interpretation of results, or preparation of manuscripts or abstracts.

Data Sharing and Competing Interests

We are committed to the sharing of final research data, being mindful that the rights and privacy of our research participants must be protected at all times, that there is the need to protect patentable and other proprietary data (ie, our web-based platform), and that restrictions on data sharing may be imposed by agreements with third parties. Published data with non-identifiers will also be shared with the 2018 Physical Activity Guidelines committee upon request. No authors have any competing interests.

For peer review only - http://bmjopen.bmj.com/site/about/guidelines.xhtml

Figure 1. Study Flowchart

For peer review only

For peer review only - http://bmjopen.bmj.com/site/about/guidelines.xhtml

Table 1. SUPER-HEALTH Study Design and Intervention Plan

Intervention Arm	Phase	Health Education Content	M2M Exercise Videos	Social Networking Support
**Attention Control** (Enables estimation of research personnel involvement effect [*within group*])	Adoption	Weekly	None	*Not offered*
	Transition	Biweekly	None	*Not offered*
	Maintenance	Monthly	None	*Not offered*
**M2M** (Enables estimation of home-based M2M [*between group comparison with AC*])	Adoption	Weekly	Weekly new video	*Not offered*
	Transition	Biweekly	Biweekly new video	*Not offered*
	Maintenance	Monthly	Monthly new video	*Not offered*
**M2M^{Plus}** (Enables estimation of social support of M2M^{Plus} [*between group comparison to M2M*])	Adoption	Weekly	Weekly new video	Offered
	Transition	Biweekly	Biweekly new video	Offered
	Maintenance	Monthly	Monthly new video	Offered

M2M = Movement-2-Music; M2M^{Plus} = M2M + Social Networking

For peer review only - http://bmjopen.bmj.com/site/about/guidelines.xhtml

Table 2. Intervention Fidelity Plan

Treatment Fidelity Element	Strategies
Study Design	Ensure intervention consistency with SCT.
	Protocol for eHealth platform maintenance and resolution of technical problems during study.
	Weekly delivery and notification of M2M videos to motivate participants to use.
	Phone coaching would assess the use of the eHealth platform and motivate intervention participants to complete their sessions.
	Password protection to ensure that participants access only material belonging to their study arm.
	Wearing of Fitbit will be monitored and if no data shows for 7 days, the participant will be contacted by phone and/or email.
	Intervention delivered via eHealth platform, not via clinicians.
Provider Training	Training of all individuals who will be conducting the screening, phone coaching, and interviews. Manual of Operating procedure to contain standardized scripts for all interactions. Booster training sessions conducted every six months.
	Intervention will be delivered through eHealth platform in exactly the same way to all participants of the two intervention arms.
Delivery of Intervention	Prescribed behaviors (aerobic/strength moves), as defined by the M2M protocol, performed by the interventionist in M2M videos will be qualitatively and quantitatively assessed.
	Technology acceptance will be measured through perceived usefulness and ease of use scale that will provide information about the usability and the extent to which the participant has positive perceptions and intentions to use the platform.
	The research coordinator will regularly monitor the eHealth platform throughout the study to ensure videos and other materials were working correctly.
	Technical difficulties with the delivery of the intervention will be resolved in timely manner.
	The number of participants logins, clicks to video links and minutes watched will be monitored by tools on the intervention eHealth platform server.
Receipt of Intervention	Every time a participant views a M2M video, a timestamp and minutes viewed are recorded.
	Each time the participant reads an articles or opens a video, they have a check in button they press.

For peer review only - http://bmjopen.bmj.com/site/about/guidelines.xhtml

Table 3. Measures, Instruments and Time Points

Variables	Instruments	Time Point	Role
Physical Activity[1]	Godin Leisure-Time Exercise Questionnaire[41]	B, A, T, M	Primary Outcome
Cardiorespiratory Endurance[1]	Sub-max Arm Ergometer	B, A, M	Secondary Outcome
	Spirometry		
Strength[1]	Grip Strength	B, A, M	Secondary Outcome
Adherence[1]	Tracking data	ongoing	Secondary Outcome
Secondary health conditions[2]	NIH PROMIS* questionnaires (pain, fatigue, sleep, QoL)	B, A, T, M	Secondary Outcome
Social Cognitive Theory Constructs[2]	Exercise Self-Efficacy Scale[53]	B, A, T, M	Mediation
	Exercise Goal-setting Scale[54]		
	Multidimensional Outcomes Expectations for Exercise Scale (MOEES)[55]		
	Barriers in Physical Activity Questionnaire – Mobility Impairment[56]		
	Social Provisions Scale[57]		
Demographics[2]	Questionnaire	Baseline	Covariate
Health History[2]	Questionnaire	B, A, M	Covariate
Physical Function[1]	Timed up and go, Repeated chair stands, Timed 25-foot walk	B, A, M	Secondary Outcome
Blood Pressure[1]	Blood Pressure Monitor	B, A, M	Covariate
Anthropometrics[1]	Height, Weight, BMI, Waist Circumference	B, A, M	Covariate
App quality & usability	eHealth Literacy Scale (eHEALS)	B, M	Covariate
	Systems Usability Scale (SUS), Mobile Application Rating Scale (MARS)	A	

For peer review only

REFERENCES

1. Lai B CK, Vanerbom K, Bickel C Scott, Rimmer JH, Motl RW. Characteristics of adults with neurologic disability recruited for exercise trials: a secondary analysis. *Adapted Physical Activity Quarterly* In Press doi: 10.1123/apaq.2017-0109
2. Chen AY, Kim SE, Houtrow AJ, et al. Prevalence of obesity among children with chronic conditions. *Obesity (Silver Spring, Md)* 2010;18(1):210-3. doi: 10.1038/oby.2009.185 [published Online First: 2009/06/13]
3. Fox MH, Witten MH, Lullo C. Reducing Obesity Among People With Disabilities. *Journal of disability policy studies* 2014;25(3):175-85. doi: 10.1177/1044207313494236
4. Froehlich-Grobe K, Lee J, Washburn RA. Disparities in obesity and related conditions among Americans with disabilities. *Am J Prev Med* 2013;45(1):83-90. doi: 10.1016/j.amepre.2013.02.021 [published Online First: 2013/06/26]
5. Hsieh K, Rimmer JH, Heller T. Obesity and associated factors in adults with intellectual disability. *Journal of Intellectual Disability Research* 2014;58(9):851-63. doi: 10.1111/jir.12100
6. Liou TH, Pi-Sunyer FX, Laferrere B. Physical disability and obesity. *Nutrition reviews* 2005;63(10):321-31. [published Online First: 2005/11/22]
7. Rimmer JH, Wang E. Obesity prevalence among a group of Chicago residents with disabilities. *Arch Phys Med Rehabil* 2005;86(7):1461-4. [published Online First: 2005/07/09]
8. Rimmer JH, Wang E, Yamaki K, et al. Documenting disparities in obesity and disability2010.
9. Carroll DD, Courtney-Long EA, Stevens AC, et al. Vital signs: disability and physical activity--United States, 2009-2012. *MMWR Morb Mortal Wkly Rep* 2014;63(18):407-13. [published Online First: 2014/05/09]
10. Ravesloot C, Seekins T, Young QR. Health Promotion for People with Chronic Illness and Physical Disabilities: The Connection between Health Psychology and Disability Prevention1998.
11. Rejeski JW, Focht CB. Aging and Physical Disability: On Integrating Group and Individual Counseling with the Promotion of Physical Activity. *Exercise and Sport Sciences Reviews* 2002;30(4):166-70.
12. Rejeski WJ, Brawley LR, Haskell WL. The prevention challenge: an overview of this supplement. *Am J Prev Med* 2003;25(3 Suppl 2):107-9. [published Online First: 2003/10/14]
13. Rimmer JH, Riley B, Creviston T, et al. Exercise training in a predominantly African-American group of stroke survivors. *Medicine and science in sports and exercise* 2000;32(12):1990-6. [published Online First: 2000/12/29]
14. Martin Ginis KA, Ma JK, Latimer-Cheung AE, et al. A systematic review of review articles addressing factors related to physical activity participation among children and adults with physical disabilities. *Health psychology review* 2016;10(4):478-94. doi: 10.1080/17437199.2016.1198240 [published Online First: 2016/10/30]
15. Becker H, Stuifbergen A. What Makes It So Hard? Barriers to Health Promotion Experienced by People With Multiple Sclerosis and Polio2004.
16. Scelza W, Kalpakjian C, Zemper E, et al. Perceived Barriers to Exercise in People with Spinal Cord Injury2005.
17. Phillips M, Flemming N, Tsintzas K. An exploratory study of physical activity and perceived barriers to exercise in ambulant people with neuromuscular disease compared with unaffected controls. *Clinical Rehabilitation* 2009;23(8):746-55. doi: 10.1177/0269215509334838
18. Zalewski K. Exploring Barriers to Remaining Physically Active: A Case Report of a Person with Multiple Sclerosis2007.
19. Clarke P, Ailshire JA, Bader M, et al. Mobility Disability and the Urban Built Environment. *American Journal of Epidemiology* 2008;168(5):506-13. doi: 10.1093/aje/kwn185

For peer review only - http://bmjopen.bmj.com/site/about/guidelines.xhtml

20. Vasudevan V, Rimmer JH, Kviz F. Development of the Barriers to Physical Activity Questionnaire for People with Mobility Impairments. *Disability and health journal* 2015;8(4):547-56. doi: 10.1016/j.dhjo.2015.04.007
21. Groah SL, Charlifue S, Tate D, et al. Spinal cord injury and aging: challenges and recommendations for future research. *Am J Phys Med Rehabil* 2012;91(1):80-93. doi: 10.1097/PHM.0b013e31821f70bc [published Online First: 2011/06/18]
22. Rimmer JH. Exercise and physical activity in persons aging with a physical disability. *Physical medicine and rehabilitation clinics of North America* 2005;16(1):41-56. doi: 10.1016/j.pmr.2004.06.013 [published Online First: 2004/11/25]
23. Webb TL, Joseph J, Yardley L, et al. Using the internet to promote health behavior change: a systematic review and meta-analysis of the impact of theoretical basis, use of behavior change techniques, and mode of delivery on efficacy. *Journal of medical Internet research* 2010;12(1):e4. doi: 10.2196/jmir.1376 [published Online First: 2010/02/19]
24. Cuijpers P, van Straten A, Andersson G. Internet-administered cognitive behavior therapy for health problems: a systematic review. *Journal of Behavioral Medicine* 2008;31(2):169-77. doi: 10.1007/s10865-007-9144-1
25. Rimmer JH, Lai B, Young HJ. Bending the Arc of Exercise and Recreation Technology Toward People With Disabilities. *Arch Phys Med Rehabil* 2016;97(9 Suppl):S247-51. doi: 10.1016/j.apmr.2016.02.029 [published Online First: 2016/06/09]
26. Lai B, Kim Y, Wilroy J, et al. Sustainability of exercise intervention outcomes among people with disabilities: a secondary review. *Disabil Rehabil* 2018:1-12. doi: 10.1080/09638288.2018.1432704 [published Online First: 2018/02/08]
27. Chan A, Tetzlaff JM, Altman DG, et al. Spirit 2013 statement: Defining standard protocol items for clinical trials. *Annals of Internal Medicine* 2013;158(3):200-07. doi: 10.7326/0003-4819-158-3-201302050-00583
28. Hoffmann TC, Glasziou PP, Boutron I, et al. Better reporting of interventions: template for intervention description and replication (TIDieR) checklist and guide. *BMJ (Clinical research ed)* 2014;348:g1687. doi: 10.1136/bmj.g1687 [published Online First: 2014/03/13]
29. Schulz KF, Altman DG, Moher D. CONSORT 2010 statement: updated guidelines for reporting parallel group randomised trials. *BMJ (Clinical research ed)* 2010;340:c332. doi: 10.1136/bmj.c332 [published Online First: 2010/03/25]
30. Bernet AC, Willens DE, Bauer MS. Effectiveness-implementation hybrid designs: implications for quality improvement science. *Implementation Science : IS* 2013;8(Suppl 1):S2-S2. doi: 10.1186/1748-5908-8-S1-S2
31. Curran GM, Bauer M, Mittman B, et al. Effectiveness-implementation Hybrid Designs: Combining Elements of Clinical Effectiveness and Implementation Research to Enhance Public Health Impact. *Medical care* 2012;50(3):217-26. doi: 10.1097/MLR.0b013e3182408812
32. Lindblad AS, Zingeser P, Sismanyazici-Navaie N. Incentives and barriers to neurological clinical research participation. *Clinical investigation* 2011;1(12):1663-68. doi: 10.4155/cli.11.153
33. Harris PA, Taylor R, Thielke R, et al. Research Electronic Data Capture (REDCap) - A metadata-driven methodology and workflow process for providing translational research informatics support. *Journal of biomedical informatics* 2009;42(2):377-81. doi: 10.1016/j.jbi.2008.08.010
34. Garber CE, Blissmer B, Deschenes MR, et al. American College of Sports Medicine position stand. Quantity and quality of exercise for developing and maintaining cardiorespiratory, musculoskeletal, and neuromotor fitness in apparently healthy adults: guidance for prescribing exercise. *Medicine and science in sports and exercise* 2011;43(7):1334-59. doi: 10.1249/MSS.0b013e318213fefb [published Online First: 2011/06/23]

For peer review only - http://bmjopen.bmj.com/site/about/guidelines.xhtml

35. Ferguson B. ACSM's Guidelines for Exercise Testing and Prescription 9th Ed. 2014. *The Journal of the Canadian Chiropractic Association* 2014;58(3):328-28.
36. Kietzmann JH, Hermkens K, McCarthy IP, et al. Social media? Get serious! Understanding the functional building blocks of social media. *Business Horizons* 2011;54(3):241-51. doi: https://doi.org/10.1016/j.bushor.2011.01.005
37. Ellis T, Motl RW. Physical activity behavior change in persons with neurologic disorders: overview and examples from Parkinson disease and multiple sclerosis. *Journal of neurologic physical therapy : JNPT* 2013;37(2):85-90. doi: 10.1097/NPT.0b013e31829157c0 [published Online First: 2013/05/02]
38. Bandura A. Social foundations of thought and action: a social cognitive theory. Englewood Cliffs, NJ: Prentice Hall 1986.
39. Bandura A. Health promotion by social cognitive means. *Health education & behavior : the official publication of the Society for Public Health Education* 2004;31(2):143-64. doi: 10.1177/1090198104263660 [published Online First: 2004/04/20]
40. Bandura A. Self-efficacy: The exercise of control. New York, NY, US: W H Freeman/Times Books/Henry Holt & Co. 1997.
41. Maher C, Ferguson M, Vandelanotte C, et al. A Web-Based, Social Networking Physical Activity Intervention for Insufficiently Active Adults Delivered via Facebook App: Randomized Controlled Trial. *Journal of medical Internet research* 2015;17(7):e174. doi: 10.2196/jmir.4086 [published Online First: 2015/07/15]
42. Maher CA, Lewis LK, Ferrar K, et al. Are health behavior change interventions that use online social networks effective? A systematic review. *Journal of medical Internet research* 2014;16(2):e40. doi: 10.2196/jmir.2952 [published Online First: 2014/02/20]
43. Befort CA, Donnelly JE, Sullivan DK, et al. Group versus individual phone-based obesity treatment for rural women. *Eating behaviors* 2010;11(1):11-7. doi: 10.1016/j.eatbeh.2009.08.002 [published Online First: 2009/12/08]
44. Godin Leisure-Time Exercise Questionnaire. *Medicine&amp Science in Sports &amp Exercise* 1997;29(Supplement):36-38. doi: 10.1097/00005768-199706001-00009
45. Rimmer JH, Rauworth A, Wang E, et al. A randomized controlled trial to increase physical activity and reduce obesity in a predominantly African American group of women with mobility disabilities and severe obesity. *Preventive Medicine* 2009;48(5):473-79. doi: https://doi.org/10.1016/j.ypmed.2009.02.008
46. Rimmer J, Wang E, Pellegrini C, et al. Telehealth Weight Management Intervention for Adults with Physical Disabilities A Randomized Controlled Trial2013.
47. Froehlich-Grobe K, White GW. Promoting physical activity among women with mobility impairments: a randomized controlled trial to assess a home- and community-based intervention 1 1No commercial party having a direct financial interest in the results of the research supporting this article has or will confer a benefit upon the author(s) or upon any organization with which the author(s) is/are associated. *Archives of Physical Medicine and Rehabilitation* 2004;85(4):640-48. doi: https://doi.org/10.1016/j.apmr.2003.07.012
48. Froehlich-Grobe K, Lee J, Aaronson L, et al. Exercise for Everyone: A randomized controlled trial of Project Workout On Wheels in promoting exercise among wheelchair users. *Archives of physical medicine and rehabilitation* 2014;95(1):20-28. doi: 10.1016/j.apmr.2013.07.006
49. Katherine SB, John PAI, Claire M, et al. Power failure: why small sample size undermines the reliability of neuroscience. *Nature Reviews Neuroscience* 2013;14(5):365. doi: 10.1038/nrn3475
50. Bailar JC, Iii, Mosteller F. Guidelines for statistical reporting in articles for medical journals: Amplifications and explanations. *Annals of Internal Medicine* 1988;108(2):266-73. doi: 10.7326/0003-4819-108-2-266

For peer review only - http://bmjopen.bmj.com/site/about/guidelines.xhtml

51. Saville DJ. Multiple Comparison Procedures: The Practical Solution. *The American Statistician* 1990;44(2):174-80. doi: 10.2307/2684163
52. Saville DJ. Multiple Comparison Procedures—Cutting the Gordian Knot. *Agronomy Journal* 2015;107(2):730-35. doi: 10.2134/agronj2012.0394
53. P Burnham K, R Anderson D. Multimodel Inference: understanding AIC and BIC in Model Selection2004.
54. Burnham K AD. Model selection and multimodel inference: A practical information-theoretic approach. 2 ed: Springer-Verlag 2002.
55. Rimmer JH, Riley B, Wang E, et al. Physical activity participation among persons with disabilities: barriers and facilitators. *Am J Prev Med* 2004;26(5):419-25. doi: 10.1016/j.amepre.2004.02.002 [published Online First: 2004/05/29]
56. Salisbury SK, Choy NL, Nitz J. Shoulder pain, range of motion, and functional motor skills after acute tetraplegia. *Arch Phys Med Rehabil* 2003;84(10):1480-5. [published Online First: 2003/10/31]
57. Rimmer JH. The conspicuous absence of people with disabilities in public fitness and recreation facilities: lack of interest or lack of access? *American journal of health promotion : AJHP* 2005;19(5):327-9, ii. [published Online First: 2005/05/18]
58. Rimmer JH, Padalabalanarayanan S, Malone LA, et al. Fitness facilities still lack accessibility for people with disabilities. *Disability and Health Journal* 2017;10(2):214-21. doi: https://doi.org/10.1016/j.dhjo.2016.12.011
59. Cook KF, Molton IR, Jensen MP. Fatigue and aging with a disability. *Arch Phys Med Rehabil* 2011;92(7):1126-33. doi: 10.1016/j.apmr.2011.02.017 [published Online First: 2011/06/28]
60. Miriam W, Martin-Diener E, Bauer G, et al. Comparison of trial participants and open access users of a web-based physical activity intervention regarding adherence, attrition, and repeated participation. *Journal of medical Internet research* 2010;12(1):e3. doi: 10.2196/jmir.1361 [published Online First: 2010/02/12]
61. Jaarsma EA, Dijkstra PU, Geertzen JH, et al. Barriers to and facilitators of sports participation for people with physical disabilities: a systematic review. *Scandinavian journal of medicine & science in sports* 2014;24(6):871-81. doi: 10.1111/sms.12218 [published Online First: 2014/04/16]
62. Rimmer JH, Rubin SS, Braddock D. Barriers to exercise in African American women with physical disabilities. *Arch Phys Med Rehabil* 2000;81(2):182-8. [published Online First: 2000/02/11]
63. Rimmer JH, Silverman K, Braunschweig C, et al. Feasibility of a health promotion intervention for a group of predominantly African American women with type 2 diabetes. *The Diabetes educator* 2002;28(4):571-80. doi: 10.1177/014572170202800411 [published Online First: 2002/09/13]
64. Rimmer JH, Wang E, Smith D. Barriers associated with exercise and community access for individuals with stroke. *Journal of rehabilitation research and development* 2008;45(2):315-22. [published Online First: 2008/06/21]

For peer review only

For peer review only - http://bmjopen.bmj.com/site/about/guidelines.xhtml

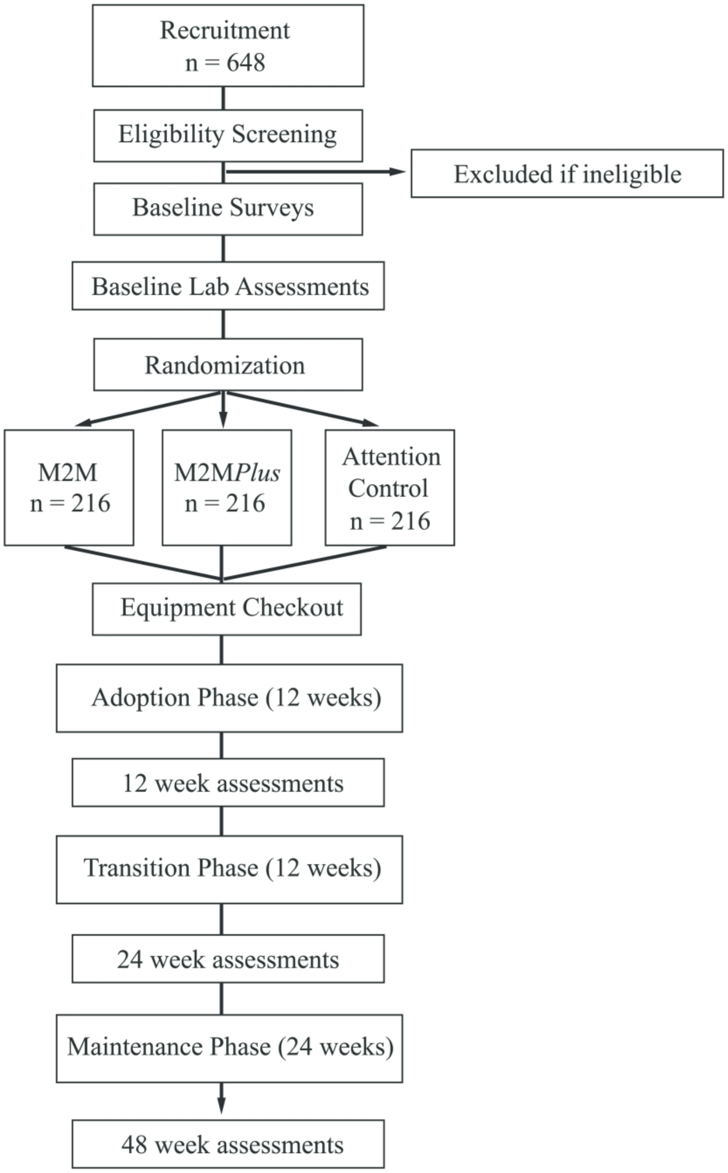

Protocol Flowchart

224x321mm (300 x 300 DPI)

The TIDieR (Template for Intervention Description and Replication) Checklist*:

Information to include when describing an intervention and the location of the information

Item number	Item	Where located ** Primary paper (page or appendix number)	Other † (details)
	**BRIEF NAME**		
**1.**	Provide the name or a phrase that describes the intervention.	Title Page	
	**WHY**		
**2.**	Describe any rationale, theory, or goal of the elements essential to the intervention.	1-5	
	**WHAT**		
**3.**	Materials: Describe any physical or informational materials used in the intervention, including those provided to participants or used in intervention delivery or in training of intervention providers. Provide information on where the materials can be accessed (e.g. online appendix, URL).	8-9	
**4.**	Procedures: Describe each of the procedures, activities, and/or processes used in the intervention, including any enabling or support activities.	10-11	
	**WHO PROVIDED**		
**5.**	For each category of intervention provider (e.g. psychologist, nursing assistant), describe their expertise, background and any specific training given.	11-12	
	**HOW**		
**6.**	Describe the modes of delivery (e.g. face-to-face or by some other mechanism, such as internet or telephone) of the intervention and whether it was provided individually or in a group.	10-12	
	**WHERE**		
**7.**	Describe the type(s) of location(s) where the intervention occurred, including any necessary infrastructure or relevant features.	Title, 18	

TIDieR checklist

For peer review only

	**WHEN and HOW MUCH**		
**8.**	Describe the number of times the intervention was delivered and over what period of time including the number of sessions, their schedule, and their duration, intensity or dose.	9-11	
	**TAILORING**		
**9.**	If the intervention was planned to be personalised, titrated or adapted, then describe what, why, when, and how.	10	
	**MODIFICATIONS**		
**10.‡**	If the intervention was modified during the course of the study, describe the changes (what, why, when, and how).	N/A	
	**HOW WELL**		
**11.**	Planned: If intervention adherence or fidelity was assessed, describe how and by whom, and if any strategies were used to maintain or improve fidelity, describe them.	13	
**12.‡**	Actual: If intervention adherence or fidelity was assessed, describe the extent to which the intervention was delivered as planned.	N/A	

** **Authors** - use N/A if an item is not applicable for the intervention being described. **Reviewers** – use '?' if information about the element is not reported/not sufficiently reported.

† If the information is not provided in the primary paper, give details of where this information is available. This may include locations such as a published protocol or other published papers (provide citation details) or a website (provide the URL).

‡ If completing the TIDieR checklist for a protocol, these items are not relevant to the protocol and cannot be described until the study is complete.

* We strongly recommend using this checklist in conjunction with the TIDieR guide (see *BMJ* 2014;348:g1687) which contains an explanation and elaboration for each item.

* The focus of TIDieR is on reporting details of the intervention elements (and where relevant, comparison elements) of a study. Other elements and methodological features of studies are covered by other reporting statements and checklists and have not been duplicated as part of the TIDieR checklist. When a **randomised trial** is being reported, the TIDieR checklist should be used in conjunction with the CONSORT statement (see www.consort-statement.org) as an extension of **Item 5 of the CONSORT 2010 Statement.** When a **clinical trial protocol** is being reported, the TIDieR checklist should be used in conjunction with the SPIRIT statement as an extension of **Item 11 of the SPIRIT 2013 Statement** (see www.spirit-statement.org). For alternate study designs, TIDieR can be used in conjunction with the appropriate checklist for that study design (see www.equator-network.org).

TIDieR checklist

For peer review only

SPIRIT

STANDARD PROTOCOL ITEMS: RECOMMENDATIONS FOR INTERVENTIONAL TRIALS

SPIRIT 2013 Checklist: Recommended items to address in a clinical trial protocol and related documents*

Section/item	Item No	Description	Addressed on page number
**Administrative information**			
Title	1	Descriptive title identifying the study design, population, interventions, and, if applicable, trial acronym	1
Trial registration	2a	Trial identifier and registry name. If not yet registered, name of intended registry	1
	2b	All items from the World Health Organization Trial Registration Data Set	1
Protocol version	3	Date and version identifier	informed consent
Funding	4	Sources and types of financial, material, and other support	19
Roles and responsibilities	5a	Names, affiliations, and roles of protocol contributors	title page
	5b	Name and contact information for the trial sponsor	19
	5c	Role of study sponsor and funders, if any, in study design; collection, management, analysis, and interpretation of data; writing of the report; and the decision to submit the report for publication, including whether they will have ultimate authority over any of these activities	18
	5d	Composition, roles, and responsibilities of the coordinating centre, steering committee, endpoint adjudication committee, data management team, and other individuals or groups overseeing the trial, if applicable (see Item 21a for data monitoring committee)	n/a

For peer review only - http://bmjopen.bmj.com/site/about/guidelines.xhtml

Introduction

Background and rationale	6a	Description of research question and justification for undertaking the trial, including summary of relevant studies (published and unpublished) examining benefits and harms for each intervention	3-5
	6b	Explanation for choice of comparators	4-5
Objectives	7	Specific objectives or hypotheses	5
Trial design	8	Description of trial design including type of trial (eg, parallel group, crossover, factorial, single group), allocation ratio, and framework (eg, superiority, equivalence, noninferiority, exploratory)	5

Methods: Participants, interventions, and outcomes

Study setting	9	Description of study settings (eg, community clinic, academic hospital) and list of countries where data will be collected. Reference to where list of study sites can be obtained	5-6
Eligibility criteria	10	Inclusion and exclusion criteria for participants. If applicable, eligibility criteria for study centres and individuals who will perform the interventions (eg, surgeons, psychotherapists)	7
Interventions	11a	Interventions for each group with sufficient detail to allow replication, including how and when they will be administered	8-12
	11b	Criteria for discontinuing or modifying allocated interventions for a given trial participant (eg, drug dose change in response to harms, participant request, or improving/worsening disease)	n/a
	11c	Strategies to improve adherence to intervention protocols, and any procedures for monitoring adherence (eg, drug tablet return, laboratory tests)	13
	11d	Relevant concomitant care and interventions that are permitted or prohibited during the trial	7
Outcomes	12	Primary, secondary, and other outcomes, including the specific measurement variable (eg, systolic blood pressure), analysis metric (eg, change from baseline, final value, time to event), method of aggregation (eg, median, proportion), and time point for each outcome. Explanation of the clinical relevance of chosen efficacy and harm outcomes is strongly recommended	13-14; table 3
Participant timeline	13	Time schedule of enrolment, interventions (including any run-ins and washouts), assessments, and visits for participants. A schematic diagram is highly recommended (see Figure)	20

For peer review only - http://bmjopen.bmj.com/site/about/guidelines.xhtml

Sample size	14	Estimated number of participants needed to achieve study objectives and how it was determined, including clinical and statistical assumptions supporting any sample size calculations	14-15
Recruitment	15	Strategies for achieving adequate participant enrolment to reach target sample size	5-6

Methods: Assignment of interventions (for controlled trials)

Allocation:

Sequence generation	16a	Method of generating the allocation sequence (eg, computer-generated random numbers), and list of any factors for stratification. To reduce predictability of a random sequence, details of any planned restriction (eg, blocking) should be provided in a separate document that is unavailable to those who enrol participants or assign interventions	12
Allocation concealment mechanism	16b	Mechanism of implementing the allocation sequence (eg, central telephone; sequentially numbered, opaque, sealed envelopes), describing any steps to conceal the sequence until interventions are assigned	11-12
Implementation	16c	Who will generate the allocation sequence, who will enrol participants, and who will assign participants to interventions	8
Blinding (masking)	17a	Who will be blinded after assignment to interventions (eg, trial participants, care providers, outcome assessors, data analysts), and how	12
	17b	If blinded, circumstances under which unblinding is permissible, and procedure for revealing a participant's allocated intervention during the trial	n/a

Methods: Data collection, management, and analysis

Data collection methods	18a	Plans for assessment and collection of outcome, baseline, and other trial data, including any related processes to promote data quality (eg, duplicate measurements, training of assessors) and a description of study instruments (eg, questionnaires, laboratory tests) along with their reliability and validity, if known. Reference to where data collection forms can be found, if not in the protocol	13-14; table 3
	18b	Plans to promote participant retention and complete follow-up, including list of any outcome data to be collected for participants who discontinue or deviate from intervention protocols	13

For peer review only - http://bmjopen.bmj.com/site/about/guidelines.xhtml

Data management	19	Plans for data entry, coding, security, and storage, including any related processes to promote data quality (eg, double data entry; range checks for data values). Reference to where details of data management procedures can be found, if not in the protocol	8
Statistical methods	20a	Statistical methods for analysing primary and secondary outcomes. Reference to where other details of the statistical analysis plan can be found, if not in the protocol	15-16
	20b	Methods for any additional analyses (eg, subgroup and adjusted analyses)	15-16
	20c	Definition of analysis population relating to protocol non-adherence (eg, as randomised analysis), and any statistical methods to handle missing data (eg, multiple imputation)	15-16
**Methods: Monitoring**			
Data monitoring	21a	Composition of data monitoring committee (DMC); summary of its role and reporting structure; statement of whether it is independent from the sponsor and competing interests; and reference to where further details about its charter can be found, if not in the protocol. Alternatively, an explanation of why a DMC is not needed	16
	21b	Description of any interim analyses and stopping guidelines, including who will have access to these interim results and make the final decision to terminate the trial	n/a
Harms	22	Plans for collecting, assessing, reporting, and managing solicited and spontaneously reported adverse events and other unintended effects of trial interventions or trial conduct	15
Auditing	23	Frequency and procedures for auditing trial conduct, if any, and whether the process will be independent from investigators and the sponsor	17
**Ethics and dissemination**			
Research ethics approval	24	Plans for seeking research ethics committee/institutional review board (REC/IRB) approval	15
Protocol amendments	25	Plans for communicating important protocol modifications (eg, changes to eligibility criteria, outcomes, analyses) to relevant parties (eg, investigators, REC/IRBs, trial participants, trial registries, journals, regulators)	15

For peer review only - http://bmjopen.bmj.com/site/about/guidelines.xhtml

Consent or assent	26a	Who will obtain informed consent or assent from potential trial participants or authorised surrogates, and how (see Item 32)	14
	26b	Additional consent provisions for collection and use of participant data and biological specimens in ancillary studies, if applicable	n/a
Confidentiality	27	How personal information about potential and enrolled participants will be collected, shared, and maintained in order to protect confidentiality before, during, and after the trial	16
Declaration of interests	28	Financial and other competing interests for principal investigators for the overall trial and each study site	19
Access to data	29	Statement of who will have access to the final trial dataset, and disclosure of contractual agreements that limit such access for investigators	16-17
Ancillary and post-trial care	30	Provisions, if any, for ancillary and post-trial care, and for compensation to those who suffer harm from trial participation	informed consent
Dissemination policy	31a	Plans for investigators and sponsor to communicate trial results to participants, healthcare professionals, the public, and other relevant groups (eg, via publication, reporting in results databases, or other data sharing arrangements), including any publication restrictions	16
	31b	Authorship eligibility guidelines and any intended use of professional writers	16
	31c	Plans, if any, for granting public access to the full protocol, participant-level dataset, and statistical code	n/a
**Appendices**			
Informed consent materials	32	Model consent form and other related documentation given to participants and authorised surrogates	appendix
Biological specimens	33	Plans for collection, laboratory evaluation, and storage of biological specimens for genetic or molecular analysis in the current trial and for future use in ancillary studies, if applicable	n/a

*It is strongly recommended that this checklist be read in conjunction with the SPIRIT 2013 Explanation & Elaboration for important clarification on the items. Amendments to the protocol should be tracked and dated. The SPIRIT checklist is copyrighted by the SPIRIT Group under the Creative Commons "Attribution-NonCommercial-NoDerivs 3.0 Unported" license.

For peer review only - http://bmjopen.bmj.com/site/about/guidelines.xhtml
